# An Integrated Engineering Approach to Intensify the Biocatalytic Metaraminol Synthesis

**DOI:** 10.1002/cssc.202502108

**Published:** 2026-02-13

**Authors:** Berit Rothkranz, Nina Klos, William Graf von Westarp, Doris Hahn, Thomas Classen, Laura Grabowski, Rocco Gentile, Jesko Kaiser, Stephan Schott‐Verdugo, Holger Gohlke, Andreas Jupke, Dörte Rother

**Affiliations:** ^1^ Institute of Bio‐ and Geosciences (IBG‐1): Biotechnology Forschungszentrum Jülich GmbH Jülich Germany; ^2^ Aachen Biology and Biotechnology (ABBt) RWTH Aachen University Aachen Germany; ^3^ Bioeconomy Science Center (BioSC) c/o Forschungszentrum Jülich GmbH Jülich Germany; ^4^ Fluid Process Engineering (AVT.FVT) RWTH Aachen University Aachen Germany; ^5^ BASF SE Ludwigshafen Germany; ^6^ Institute for Pharmaceutical Chemistry Heinrich Heine University Düsseldorf Düsseldorf Germany; ^7^ Institute of Bio‐ and Geosciences (IBG‐4): Bioinformatics Forschungszentrum Jülich GmbH Jülich Germany; ^8^ Institute of Bio‐ and Geosciences (IBG‐2): Plant Sciences Forschungszentrum Jülich GmbH Jülich Germany

**Keywords:** amine transaminase, chiral amino alcohol, continuous product extraction, oxazolidine, Pictet–Spengler product

## Abstract

Metaraminol is a chiral amino alcohol and plays an important role as a precursor molecule and active pharmaceutical ingredient in industry. Its enzymatic synthesis has been developed in recent years and can serve as an alternative to conventional synthesis routes that use toxic, fossil‐based resources. Although the enzymatic two‐step reaction toward metaraminol has been intensively investigated in the past, full conversion has never been reached in the amine transaminase‐catalyzed step. In this study, we focus on identifying and overcoming the hurdles of the transamination step to reach higher metaraminol yields. Photometric and LC‐MS analyses revealed side‐product formation as a major drawback for the enzymatic metaraminol synthesis. Besides the oxidation of (*R*)‐3‐OH‐PAC as well as its imine formation with isopropylamine, we demonstrate for the first time the adduct formation of the cofactor pyridoxal‐5’‐phosphate with metaraminol. Only by changing the amine transaminase formulation to purified enzyme and increasing the concentration by tenfold, >99% product yield with a metaraminol concentration of 75 mM was reached. Further, we successfully integrated the amine donor l‐alanine by applying a continuous product extraction system as an alternative to isopropylamine. We believe that our findings and optimization strategies can also serve as a blueprint for other amine‐based syntheses.

## Graphical abstract

The amine transaminase‐catalyzed amination of (*R*)‐3‐hydroxyphenylacetylcarbinol toward metaraminol involves severe side‐product formation, hampering high product titers. The application of a continuous extraction system together with an increased amine transaminase concentration and optimal enzyme formulation enhanced the overall metaraminol yield.

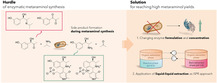

## 1 Introduction

**SCHEME 1:**
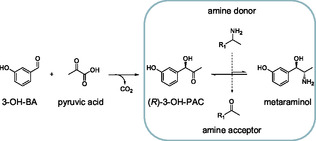
Two‐step enzymatic metaraminol synthesis. The first step comprises the decarboxylation of pyruvic acid and subsequent carboligation of the resulting acetaldehyde with 3‐hydroxybenzaldehyde (3‐OHBA) catalyzed by a pyruvate decarboxylase variant of *Acetobacter pasteurianus* (*Ap*PDC). In the second step, the amination, an amino group is transferred from an amine donor (e.g., L‐alanine or isopropylamine) to the intermediate product (*R*)‐3‐hydroxyphenylacetylcarbinol ((*R*)‐3‐OH‐PAC), resulting in the target product metaraminol. The amination is catalyzed by the wild‐type amine transaminase from *Chromobacterium violaceum* (*Cv*ATA). R_1_: –COOH; –CH_3_.

3‐[(1*R*,2*S*)‐2‐amino‐1‐hydroxypropyl]phenol, better known as metaraminol, can interact and stimulate *α*1 receptors of vascular smooth muscles, which leads to an increase in blood pressure [1, 2]. Therefore, metaraminol is a key active pharmaceutical ingredient (API) in hypotension treatment. Furthermore, it can be used as a building block for the synthesis of compounds containing the tetrahydroisoquinoline motif (THIQs), which have antitumor, antiparasitic, or anticholinergic effects [3, 4, 5, 6]. To the best of our knowledge, its current production predominantly relies on the use of metal catalysts, toxic solvents such as dichloromethane, and fossil‐based raw materials [7, 8, 9], exhibiting limited resource efficiency. This is a critical point, as the chemical industry is a growing industry sector with a major impact on global greenhouse gas emissions, for which alternative defossilized pathways are required [10]. As a more environmentally favorable and less complex alternative, a two‐step enzymatic synthesis route has been investigated in previous studies [11, 12, 13]. This enzymatic two‐step reaction starts with the carboligation of 3‐hydroxybenzaldehyde and an acyl donor, e.g., pyruvate or acetaldehyde, toward (*R*)‐3‐hydroxyphenylacetylcarbinol ((*R*)‐3‐OH‐PAC), catalyzed by a pyruvate decarboxylase variant from *Acetobacter pasteurianus* (*Ap*PDC) [13, 14, 15]. After a subsequent purification step of the intermediate product, (*R*)‐3‐OH‐PAC is applied as substrate for the second step. Here, the amine transaminase from *Chromobacterium violaceum* (*Cv*ATA) is used for transferring the amine group of a donor molecule, e.g., isopropylamine (IPA) or L‐alanine, to the substrate (*R*)‐3‐OH‐PAC, resulting in the target product metaraminol (Scheme 1) [12]. Like other amine transaminases, this fold type I amine transaminase is dependent on the cofactor pyridoxal‐5’‐phosphate (PLP), which is self‐regenerated by *Cv*ATA in the reaction mechanism, thus eliminating the need for external cofactor regeneration [16]. PLP is not only crucial for the catalytic cycle, but it is also important for the stability of *Cv*ATA [17].

While it has been shown by Labib et al. that the overall reaction scheme using biocatalysts and second‐generation feedstocks is feasible [12] and the carboligation step can already be conducted with high yield and selectivity [18, 19], the transamination step remains challenging. Within the transamination reaction, the choice of the amine donor is decisive. As mentioned above, two commonly applied ones are isopropylamine (IPA) or L‐alanine. IPA is an inexpensive bulk chemical and can be readily obtained, but it is often based on fossil resources and exhibits certain toxic properties, especially concerning aquatic organisms [20]. Although the reaction equilibrium is heavily lying on the substrate side when applying IPA, its co‐product, acetone, is volatile and can conveniently be removed by evaporation, thereby pushing the reaction equilibrium in favor of the desired product [21]. When IPA is employed, systematic investigations, especially with the substrate (*R*)‐3‐OH‐PAC, are still needed regarding co‐substrate concentration, potential side reactions, and enzyme dosage, all of which have a direct impact on process stability and yield. In contrast, L‐alanine is a nontoxic, bio‐based alternative that avoids polluting by‐products, but its transamination reactions are, like IPA, governed by an unfavorable thermodynamic equilibrium, creating the need for an additional in situ product removal (ISPR) strategy [22, 23, 24]. To address this challenge, an ISPR strategy via liquid–liquid extraction (LLE) has been proposed [11, 13], allowing immediate removal of the product from the reaction medium and thereby allowing for higher product yield. Previous studies of Doeker and Grabowski et al. concentrated on establishing and optimizing physical and reactive extraction methods using oleic acid as a reactive extractant [11, 13]. So far, the best results in the equilibrium stage were achieved by reactive extraction. However, complete utilization of the primary substrate has not been achieved so far, diminishing the productivity of the process. In this study, we pursue a fully continuous operation mode to maximize substrate conversion and unlock the full potential of the process. For this, a complete characterization of the enzymatic metaraminol synthesis starting from the amination of (*R*)‐3‐OH‐PAC is first performed, aiming to identify possible limitations and strategies for process intensification to maximize the yield, selectivity, and titer.

## 2 Results and Discussion

### 2.1 Reaction Engineering Strategies for Metaraminol Synthesis Using Isopropylamine as Amine Donor

**FIGURE 1:**
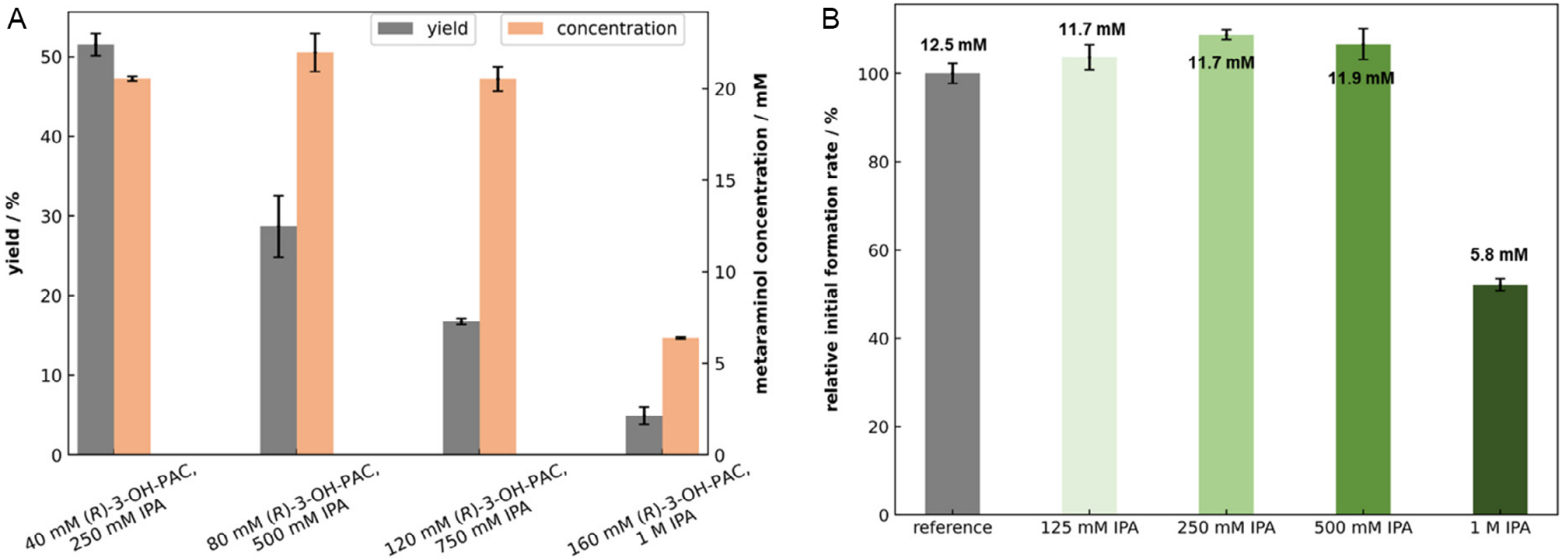
Influence of the different substrate and amino donor concentrations on the amination reaction. (A) Metaraminol yield and concentration after 24 h at different (*R*)‐3‐OH‐PAC concentrations ranging from 40 to 160 mM with 6.25‐fold excess of isopropylamine (IPA). (B) Relative initial metaraminol formation rate after enzyme incubation with IPA concentrations ranging from 125 mM to 1 M and 1 mM PLP for 3 h. Reaction is afterwards performed with an (*R*)‐3‐OH‐PAC concentration of 20 mM, 125 mM IPA, and 1 mM PLP. The reference is incubated only in KPi buffer (pH 7.5) with 1 mM PLP for 3 h. All experiments were conducted in 100 mM KPi buffer (pH 7.5) with 1 mM PLP and 10 mg mL^1^ whole cell *Cv*ATA as catalyst. T = 30°C, 850 rpm, V = 1 mL, *t* = 24 h. *n* = 2, mean ± SD.

It has been suggested that the easiest and most affordable enzyme formulation for a biocatalytic process is using lyophilized whole cells [25]. Therefore, this formulation was selected first. To evaluate the influence of the (*R*)‐3‐OH‐PAC concentration, the substrate was increased stepwise from 40 to 160 mM with a 6.25‐fold excess of IPA. A maximum of 20 mM metaraminol was produced with increasing substrate concentration from 40 to 120 mM, while with 160 mM (*R*)‐3‐OH‐PAC, the final metaraminol concentration was only 7 mM. Therewith, the yield continuously decreases with increasing substrate concentration from 50% to 5% (Figure 1A). This result hints at a possible substrate inhibition, which is frequently observed in organic synthesis with amine transaminases [17]. This possible inhibition was tested by incubating the substrate (*R*)‐3‐OH‐PAC with *Cv*ATA and its cofactor PLP for 3 h and then carrying out a reaction to measure its residual product formation rate. Already at a concentration of 40 mM (*R*)‐3‐OH‐PAC, a decrease in the product formation rate can be detected (Figure S6). This effect is strengthened with increasing substrate concentrations. Since IPA, as an amine donor, can also have an inhibitory effect on amine transaminases [26], its influence was investigated as well. An IPA concentration lower than 1 M has virtually no influence on the product formation rate, whereas at 1 M IPA, the product formation rate also decreases. It has been suggested that during the catalytic cycle, IPA can react with the enzyme–PLP complex to form enzyme‐pyridoxamine 5’‐phosphate (PMP), leading to the dissociation of the cofactor and ultimately inactivating the enzyme [27]. The inactivation could explain the significant drop in the product concentration with 160 mM (*R*)‐3‐OH‐PAC and 1 M IPA (Figure 1B). The overlapping inhibitory effects further reduce product formation. To increase the total titer, feeding could be a solution to overcome the potential substrate inhibition. However, also with substrate feeding, no higher metaraminol concentration than 20 mM was achieved (Figure S7). As the metaraminol synthesis is an equilibrium reaction, the equilibrium can also be the limiting factor. Therefore, feeding of the co‐substrate IPA and the application of a vacuum to remove the by‐product acetone were tested to further shift the equilibrium (Figure S7). This also had no positive effect on achieving higher metaraminol concentrations than 20 mM. The potential substrate inhibition alone is therefore not the only factor that causes the product limitation.

### 2.2 Identification of Side Product Formation

#### 2.2.1 Adduct Formation between Metaraminol and PLP

**FIGURE 2:**
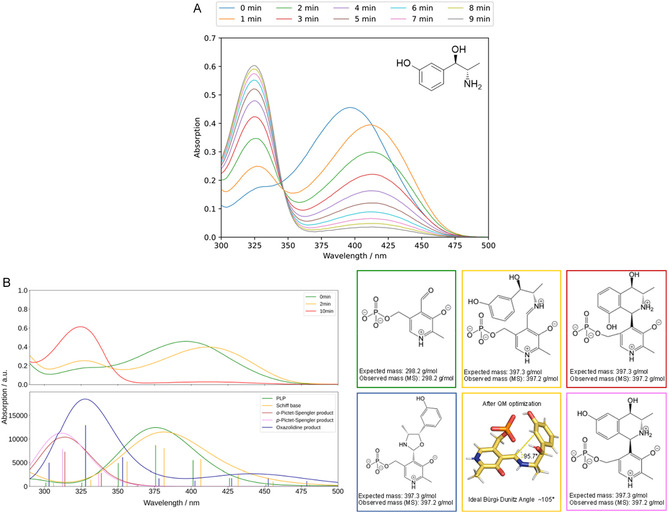
(A) Absorption spectrum of 0.1 mM PLP in the presence of 20 mM metaraminol. A PLP stock solution in 100 mM KPi buffer (pH 7.6) was prepared in a quartz glass cuvette. A metaraminol stock solution was added to the PLP solution so that the PLP and metaraminol concentration was adjusted to 1 and 10 mM, respectively. Immediately after the addition of metaraminol, wavelength scans of this reaction mixture were recorded every minute for 10 min in total, ranging from *λ* = 300–500 nm. As a reference, a 0.1 mM PLP solution was measured in the same way. (B) Experimental (above) and computed UV–vis spectra (below) identify the oxazolidine and the Pictet–Spengler product. The computed spectra for PLP (green), Schiff base (orange), the Pictet–Spengler products (brown for the product resulting from the *ortho*‐attack, pink for the one from the *para*‐attack), and the oxazolidine (dark blue) were obtained as described in the supporting materials and methods (Supporting Information). The 2D structures of the compounds are reported together with the expected and observed masses (calculated with mass spectrometry). The reported stereoisomers and protonation states are the ones that lead to the best agreement between computed and experimental spectra. The different protonation states and stereoisomers considered are reported Figure S13 and Table S1.

The cofactor PLP is crucial for the catalytic cycle as well as for the stability of amine transaminases and is therefore commonly added to the reaction mixtures [28, 29, 30]. Interestingly, during the preparation of a 20 mM metaraminol solution containing 0.1 mM PLP, the typical yellow color of PLP disappeared within a very short time (∼10 min), and the solution became colorless. Therefore, photometric studies of 0.1 mM PLP as a single substance and 0.1 mM PLP in the presence of 20 mM metaraminol were performed in the absence of enzyme (Figure 2A).

The results demonstrate that in the presence of metaraminol, the original absorption maximum at approx. 390 nm shifts to a wavelength of 410 nm after one minute, in line with the formation of an aldimine Schiff base [31]. Additionally, the peak almost disappears within 10 min, with the concomitant formation of a new absorption maximum at 325 nm, which could indicate the formation of pyridoxamine‐phosphate (PMP) [32]. This does not occur when solely incubating 0.1 mM PLP in 100 mM KPi buffer (Figure S8A). When investigating the absorption spectrum of PLP in the presence of a similar chiral amino alcohol (1*S*,2*R*)‐norephedrine, the initial shift from 390 to 410 nm can also be observed, which could indicate the formation of a Schiff base [32]. However, the new absorption peak remains unchanged, and no new absorption maximum is formed at 325 nm (Figure S8B). To clarify which compound is generated when PLP is incubated in the presence of metaraminol, liquid chromatography/mass spectrometry (LC‐MS) measurements were performed. Here, the results confirmed the formation of a complex between metaraminol and PLP with an m/z 397.2 [M+H]^+^ (Figure S11), which either fits the Schiff base, an oxazolidine compound, or a Pictet–Spengler product of both components.

Nevertheless, it is difficult to unambiguously identify the origin of the side product, as they are potentially unstable under different reaction conditions. To further assess the possible byproducts, quantum mechanics computations were performed. The result of the simulated absorption spectra support that the measured absorption spectrum of PLP in the presence of metaraminol can originate from the oxazolidine compound or the Pictet–Spengler product (Figure 2B).

For that, after the condensation to the Schiff base, a ring closure occurs following either a 5‐exo‐trig [33] or a 6‐endo‐trig reaction [34]. Considering the Pictet–Spengler product in a neutral medium (pH 7.4), two possible regioisomers of the product could be formed, the *ortho*‐ and the *para*‐product (Figure 2B), depending on the position of the phenolic hydroxy group of metaraminol with respect to the attacking position of the electron‐rich aromatic system to the electrophilic iminium ion [35, 36]. The attack‐optimized Schiff structures reveal an angle that falls within a plausible range from the ideal Bürgi–Dunitz attack angle [37]. This occurs only when the phenolic acid of the metaraminol sub‐moiety is present in the *ortho*‐position with respect to the imine attack with subsequent ring closure (Figures 2B, Figure S12). The ideal attack angle does not fit optimally for the oxazolidine‐like ring closure either (Figure S12). According to the computed spectra, both products have similar absorption maxima. Multiple protonation states and region‐stereoisomers were considered (Table S1 and Figure S13). Pictet–Spengler *para*‐products are generally more preferred in water [6, 38, 39, 40, 41, 42, 43], but the regioselectivity can change depending on the reaction conditions [44]. The best fitting protonation state/regio‐stereoisomer to the experimental spectra was selected. The oxazolidine also has a similar absorption maximum. Therefore, we cannot exclude the presence of all three derivatives in solution.

Based on the newly gained insights, it was reasonable to assume that the PLP added at the beginning of the reaction would react directly with the produced metaraminol to form the Pictet–Spengler product, rendering it unavailable for cofactor exchange as the *Cv*ATA exhibits a low PLP binding affinity compared to other amine transaminases [29]. The effect of adding PLP at different time points during the reaction on the metaraminol concentration was therefore tested. However, feeding the cofactor did not lead to enhanced titers, as the metaraminol concentration remained comparably low at 3 mM after 24 h (Figure S14). Besides the reaction of PLP with metaraminol, other side‐products were detected during the amination, which could also influence the reaction toward metaraminol.

#### 2.2.2 (*R*)‐3‐OH‐PAC Forms Various Side‐Products

**FIGURE 3:**
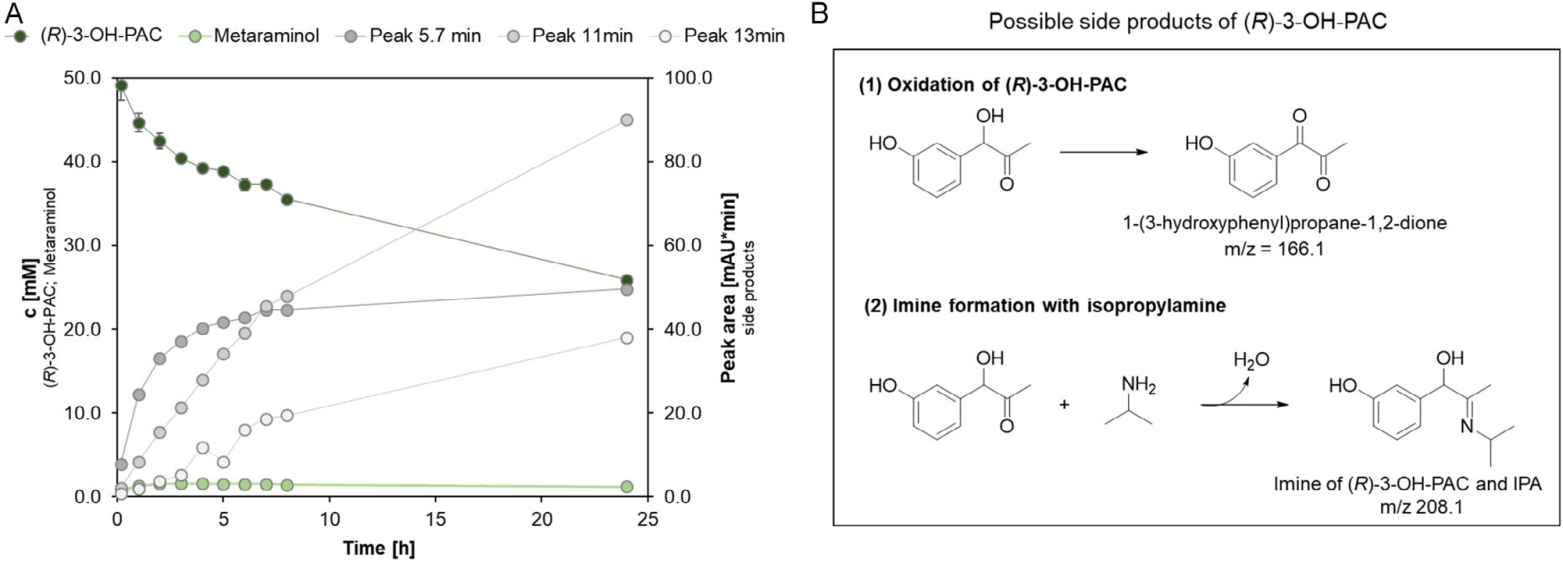
(A) Side product and metaraminol formation over the reaction time. Reaction conditions: 20 mg·mL^−1^ whole cell *Cv*ATA, 40 mM (*R*)‐3‐OH‐PAC, 125 mM IPA, and 1 mM PLP in 100 mM KPi buffer (pH 7.6). V = 1 mL, T = 30°C, 850 rpm. *n* = 2, mean ± SD. (B) Oxidation of the substrate (*R*)‐3‐OH‐PAC to 1‐(3‐hydroxyphenyl)propane‐1,2‐dione and formation of an imine between (*R*)‐3‐OH‐PAC and the amine donor IPA.

During some experiments using whole cell *Cv*ATA, little to no metaraminol synthesis could be detected, although the (*R*)‐3‐OH‐PAC concentration decreased. New peaks appeared in the high performance liquid chromatography (HPLC) chromatogram at retention times of 5.7 min, 11 min, and 13 min (*λ* = 277 nm) and increased in proportion to the decreasing substrate (*R*)‐3‐OH‐PAC (Figure 3A). Subsequent LC‐MS analysis of the reaction samples revealed an m/z 166.1 [M+H]^+^ for the side‐product peak at 11 min (Figure S15). This could hint at the oxidation product of (*R*)‐3‐OH‐PAC, 1‐(3‐hydroxyphenyl)propane‐1,2‐dione, which is depicted in Figure 3B. As the oxidation product, 1‐(3‐hydroxyphenyl)propane‐1,2‐dione, potentially could be a substrate for the *Cv*ATA, further side‐products could be formed. The LC‐MS analysis also detected a precursor mass of 208.1 Da of the component eluting at 13 min, potentially being the imine of (*R*)‐3‐OH‐PAC and the amine donor IPA (Figure S16). The previous results indicate that the biocatalytic metaraminol synthesis suffers from severe side‐product formation, hampering high yields under the conditions evaluated above. Additionally, it seems that the side‐product formation proceeds faster than the metaraminol synthesis (Figure 3A), which leads to the question of whether an increasing biocatalyst concentration can foster the metaraminol formation.

### 2.3 Overcoming the Metaraminol Production Limit by Changing *Cv*ATA Concentration and Formulation

**FIGURE 4:**
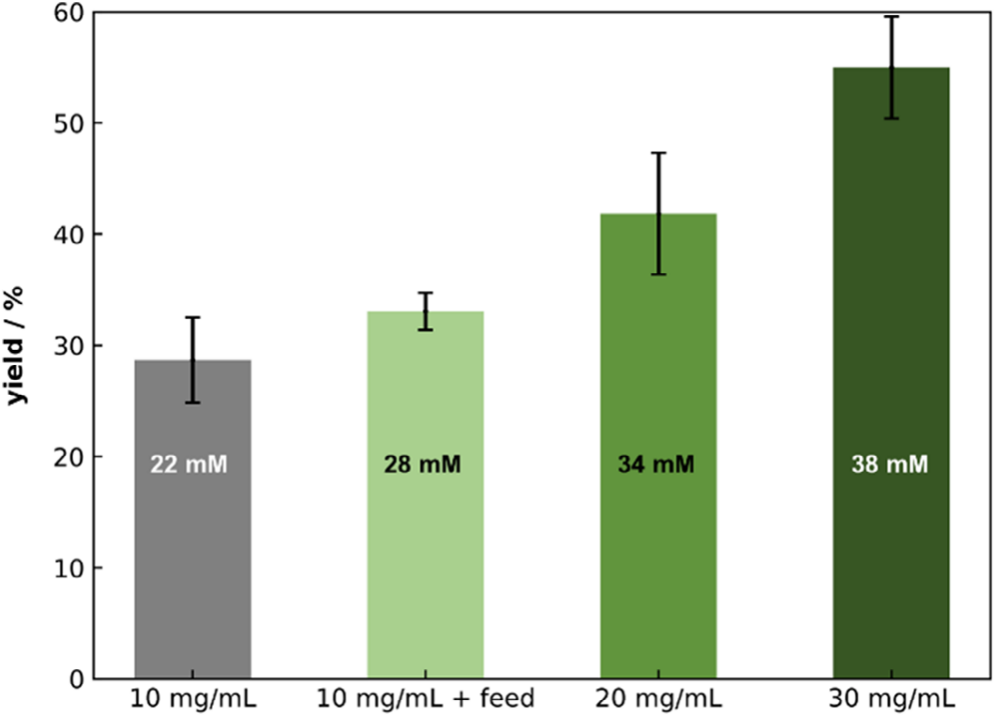
Metaraminol yield and concentration after 24 h, increasing the whole cell catalyst load. Various lyophilized whole cell *Cv*ATA concentrations were used: 10 mg·mL^−1^, initial 10 mg·mL^−1^and after 5 h feed of 10 mg·mL^−1^, initial 20 mg·mL^−1^, and initial 30 mg·mL^−1^. All experiments were conducted using 80 mM (*R*)‐3‐OH‐PAC, 500 mM IPA, 1 mM PLP in 100 mM KPi buffer (pH 7.5), T = 30°C, 850 rpm, V = 1 mL, *t* = 24 h. *n* = 2, mean ± SD.

To overcome the production limit of so far ∼20 mM metaraminol, different *Cv*ATA concentrations and formulations were tested. In the previous chapter, we showed that the side‐product formation based on (*R*)‐3‐OH‐PAC directly competes with the amination of (*R*)‐3‐OH‐PAC toward metaraminol. To push the reaction in the direction of our target product, metaraminol, we first increased the concentration of lyophilized whole cells harboring the *Cv*ATA as a catalyst. The expectation was that the conversion toward metaraminol would be faster than the spontaneous oxidation of the (*R*)‐3‐OH‐PAC over time. The metaraminol yield and the final concentration after 24 h using whole cell concentrations ranging from 10 mg·mL^−1^ to 30 mg·mL^−1^ are depicted in Figure 4. The results indicate that an increase in the concentration of added biocatalyst corresponds to an increase in the production of metaraminol. In the case of 30 mg·mL^−1^ whole cells, the highest metaraminol concentration with 38 mM was reached, which is equal to a metaraminol yield of approx. 50% related to the initial substrate concentration. By using this whole cell concentration, it was possible to increase the synthesized metaraminol concentration by 2‐fold compared to the prior applied whole cell catalyst load of 10 mg·mL^−1^. These results support the hypothesis that the noncatalyzed by‐product formation proceeds faster than the target reaction to metaraminol under the previously applied reaction parameters. Since high concentrations of whole cells increase the viscosity of the reaction solution and thus impair mass transfer in the system [22, 45], the concentration of whole cells cannot be increased until complete conversion is achieved. Additionally, whole cells can retain the product, which increases the number of downstream processing steps and could lead to product loss.

**FIGURE 5:**
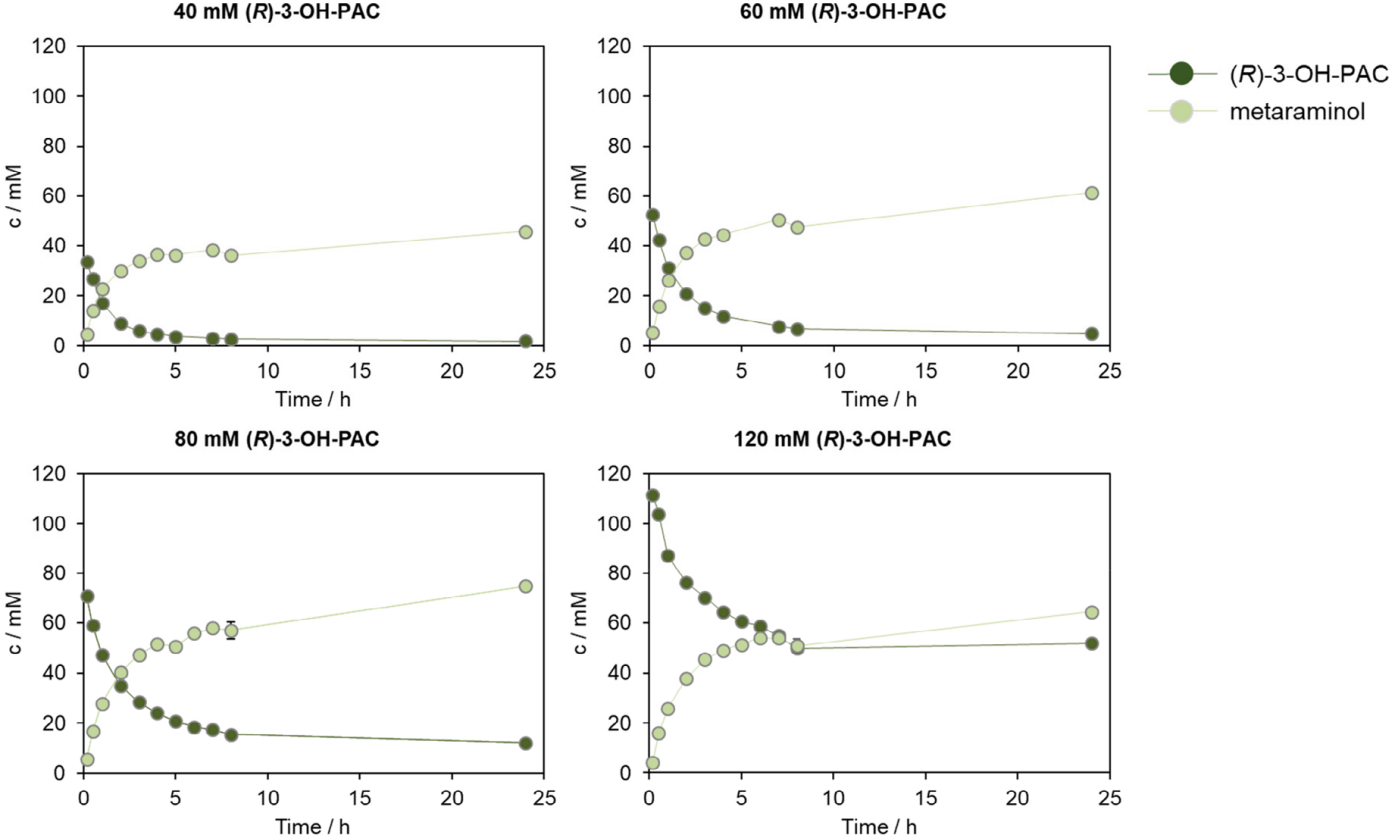
Metaraminol formation and (*R*)‐3‐OH‐PAC depletion using 6 mg·mL^−1^ purified *Cv*ATA at various substrate concentrations ranging from 40–120 mM. Reaction conditions: 10 mg·mL^−1^ lyophilized and purified *Cv*ATA, 40–120 mM (*R*)‐3‐OH‐PAC, 6.25‐fold excess of IPA, and 1 mM PLP in 100 mM KPi buffer (pH 7.6). V = 1 mL, 850 rpm, T = 30°C, *t* = 24 h. *n* = 2, mean ± SD.

Therefore, we changed the *Cv*ATA formulation from whole cells to purified, lyophilized *Cv*ATA. In Figure 5, varying (*R*)‐3‐OH‐PAC concentrations from 40 to 120 mM were tested using a constant *Cv*ATA concentration of 6 mg·mL^−1^. With 40 mM (*R*)‐3‐OH‐PAC, we indeed achieved full conversion (>99%) after 24 h. Up to an (*R*)‐3‐OH‐PAC concentration of 80 mM, a metaraminol yield of >95% was reached with the highest absolute metaraminol concentration of 75 mM after 24 h. Interestingly, with an initial (*R*)‐3‐OH‐PAC concentration of 120 mM, a maximum of 64 mM metaraminol was reached, which refers to a yield of 53%. These results go hand in hand with those described in the previous chapters, where different substrate loads were compared using whole cell catalysts (Figure 1) and a possible substrate inhibition was discussed.

All in all, using 60 mM (*R*)‐3‐OH‐PAC as the initial substrate is the sweet spot between a high metaraminol yield (>99%) and a high absolute metaraminol concentration of 61  ± 0.6 mM. Although whole cell biocatalysts have the advantage of being produced faster and having lower process costs due to the lack of enzyme purification [25], the application of purified enzymes in the metaraminol synthesis allows for higher catalyst concentrations, which can be adjusted to reach full conversion without increasing the viscosity of the reaction solution and minimizing side‐product formation. However, as purified enzyme in such high concentrations can be very expensive in a process, the costs could be reduced by immobilization and subsequent reuse of the *Cv*ATA, as already shown by other working groups [46, 47].

### 2.4 Continuous Processing With ISPR to Improve Yields with L‐Alanine as Amine Donor

**FIGURE 6:**
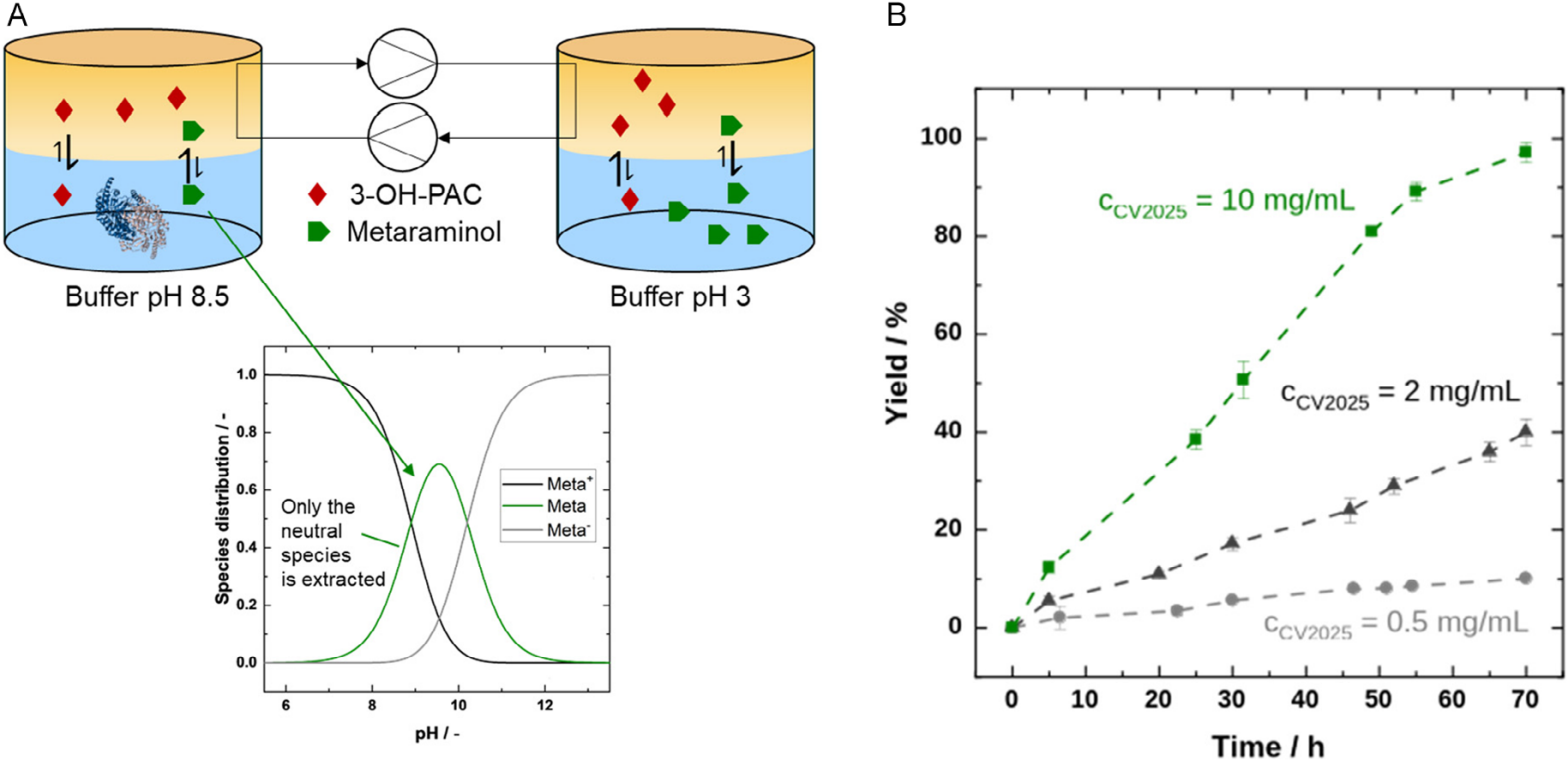
(A) Schematic depiction of the continuous LLE system with respect to the species distribution of metaraminol according to Mack et al. [11]. (B) Metaraminol yield over the reaction time of 70 h using a continuous LLE system. The aqueous reaction phase (7 mL 100 mM KPi buffer, pH 8.5) contained 0.5, 2, or 10 mg·mL^−1^ purified and lyophilized *Cv*ATA, 250 mM L‐alanine, and 1 mM PLP. The organic phase consisted of 20 mL of 1‐octanol saturated with 30 mM (*R*)‐3‐OH‐PAC, which was transferred between the 20 mL glass vials at a pumping rate of 2 mL·min^−1^. Metaraminol was extracted with an acidic aqueous solution (pH 3.0) with 250 mM HCl. *n* = 2, mean ± SD.

As IPA is a fossil‐based bulk chemical, the amine donor was changed to the more sustainable alternative L‐alanine, combined with an in situ extraction approach. The continuous experiments were conducted for 70 h, featuring simultaneous forward and back extraction (Figure 6A). Different enzyme concentrations (0.5, 2, and 10 mg·mL^−1^) were evaluated under moderate pump rates (2 mL·min^−1^). Figure 6B illustrates the conversion over time for these three enzyme concentrations, as measured from the back‐extraction phase. In all three cases, the conversion increases almost linearly, with higher catalyst loadings resulting in steeper slopes. This behavior suggests that the reaction kinetics are rate‐limiting, whereas mass transport to the organic phase and the pumping rate are sufficiently fast.

Depending on the selected catalyst load, final conversions ranging from 10% to 97% were achieved based on an initial (*R*)‐3‐OH‐PAC concentration of 30 mM. Since the slopes are still linear at the end of the experiment, it is likely that yields ≥99% would have been reached by extending the reaction time further. The highest enzyme concentration (10 mg·mL^−1^) reached 97% conversion after 70 h, whereby the conversion rate was slightly reduced toward the end of the experiment. Thus, these results demonstrate that the more sustainable co‐substrate L‐alanine, accessible from renewable resources, can also be successfully applied. Nevertheless, this continuous approach requires additional apparatus and solvent usage to achieve nearly full conversion. Notably, despite moderate pump rates (2 mL·min^−1^) and a distribution coefficient for metaraminol of only about 0.2 at pH 8.5, as reported by Doeker in 2024 [48], the extraction system was not found to be limiting. This observation indicates that further improvements, especially in enzyme kinetics, represent a logical next step. Here, enzyme engineering or a broad enzyme screening regarding higher activity toward (*R*)‐3‐OH‐PAC could be solutions to minimize the reaction time and enzyme amount. Further, the phase ratio of organic to aqueous phase can be reduced in future work in order to achieve the concentration of the target product.

## 3 Conclusion

Previous studies on the enzymatic two‐step reaction focused on the sustainable production of precursor molecules for the metaraminol synthesis as well as the integration of ISPR strategies, shifting the thermodynamic equilibrium when using L‐alanine as amine donor [11, 13]. While both approaches were successfully established, the reaction toward metaraminol still lacked full conversion of the substrate. An accumulated product yield of 69% was reached after three extraction steps over 72 h, resulting in a space‐time‐yield (STY) of 0.6 g·L^−1^· h^−1^. In this study, we aimed to enhance the overall metaraminol yield and titer, starting with the reaction system using IPA as an amine donor. Here, a metaraminol production limit of 20 mM has been identified, which we needed to overcome by characterizing and optimizing the amine transaminase‐catalyzed reaction. While the (*R*)‐3‐OH‐PAC as well as the IPA concentrations exhibited a rather low impact on the metaraminol titer, several side reactions were uncovered as the major hurdle of metaraminol synthesis. On the one hand, we revealed the formation of a Pictet–Spengler and an oxazolidine product between the cofactor PLP and the target product metaraminol. However, it remains unclear if this complex formation occurs in the active site of the *Cv*ATA and if it affects the overall enzyme stability. On the other hand, the oxidation of the substrate (*R*)‐3‐OH‐PAC in the aqueous reaction system, as well as the imine formation which directly competes with the enzymatic metaraminol synthesis. Although the use of purified enzymes is cost‐ and labor‐intensive, in this study, the application of purified *Cv*ATA led to raised metaraminol titers and yields. By increasing the purified *Cv*ATA concentration to 6 mg·mL^−1^, we improved the metaraminol titer by 3.8‐fold. Further, a STY of up to 5.2 g·L^−1^· h^−1^ was determined for the IPA reaction system, which is 8.6‐fold higher compared to the previously published approaches [13]. The final transfer of the reaction to a continuous product extraction system gave a comparable metaraminol yield of 97% after 70 h using the biobased amine donor L‐alanine, reaching a slightly increased STY of 0.7 g · L^−1^· h^−1^ compared to the previously published extraction experiments [13]. By increasing the initial (*R*)‐3‐OH‐PAC concentration, we believe that the STY could even be increased. Further investigations on the engineering of the already functioning *Cv*ATA toward a higher substrate affinity are crucial for decreasing the employed transaminase concentration while increasing the catalyst productivity.

The study presented here, along with previous publications on the subject, demonstrates that the enzymatic synthesis of metaraminol can be established. By investigating this reaction as a showcase, we were able to identify and overcome the limitations of the transaminase reaction, paving the way for testing its implementation at an industrial scale. Importantly, the findings from this study can also be transferred to the synthesis of other challenging amines or APIs. For example, compounds that require similar stereocenters as those present in metaraminol or could use metaraminol as precursors. This may facilitate the adoption of biocatalytic approaches in conventional chemical processes dealing with amine syntheses.

## 4 Experimental Section

### 4.1 Recombinant Expression of the Amine Transaminase from *Chromobacterium violaceum* (*Cv*ATA)

Chemically competent *Escherichia coli* (*E. coli*) BL21 (DE3) cells were transformed with a pET28a(+) plasmid containing the gene for the amine transaminase of *Chromobacterium violaceum* (*Cv*ATA) with an N‐terminal His_6x_‐Tag. The amino acid sequence of the *Cv*ATA can be found in the Supporting Information. Therefore, 1 µL of pET28a(+)‐His‐*Cv*ATA was transferred to 50 µL of the cell suspension and incubated for 20 min on ice. Afterwards, heat shock was performed for 60 s at 42°C. 500 µL SOC medium (3.6 g·L^−1^ glucose, 0.2 g·L^−1^ KCl, 1.0 g·L^−1^ MgCl_2_, 0.6 g·L^−1^ NaCl, 20.0 g·L^−1^ peptone, 5.0 g·L^−1^ yeast extract) was added to the cells. This cell suspension was incubated for 45 min at 37°C and 350 rpm. 50 µL were plated on LB‐agar containing 0.05 mg mL^−1^ kanamycin and stored overnight at 37°C.

A preculture was prepared from the freshly transformed *E. coli* cells by transferring a colony to 20 mL LB medium in a 100 mL baffled shake flask containing 0.05 mg·mL^−1^ kanamycin. The preculture grew overnight at 30°C and 120 rpm.

Protein production was conducted by inoculating 1 L of autoinduction medium (24 g·L^−1^ yeast extract, 12 g·L^−1^ peptone, 5 mL·L^−1^ glycerol, 0.5 g·L^−1^ glucose, 2 g·L^−1^ lactose, 90 mL·L^−1^ 1 m KPi buffer, pH 7.0) with 1 mL of the preculture in a 5 L baffled shake flask. Afterwards, the main culture was incubated at 37°C and 70 rpm. After 3 h, the temperature was reduced to 20°C to support protein folding and decrease the growth rate. After 48 h, cell harvest was performed at 7000 rpm and 10°C for 40 min using the Beckman Avanti JXN‐26 (Beckman Coulter, Brea, USA). The cells were stored at −20°C until further use. For whole cell catalyst experiments, the cells were freeze‐dried at −44°C and 0.16 mbar for ca. 24 h using the lyophilizer LyoCube from Martin Christ Gefriertrocknungsanlagen GmbH (Osterode, Germany). Expression control was performed by sodium dodecyl sulfate polyacrylamide gel electrophoresis (SDS‐PAGE), which is shown in Figure S1.

### 4.2 *Cv*ATA Purification via Immobilized Metal Affinity Chromatography (IMAC)

Prior to protein purification, a 20% (w/v) cell suspension was prepared with the recombinant *E. coli* BL21 (DE3) cells in equilibration buffer (50 mM potassium phosphate (KPi) buffer, pH 7.5, 0.2 mM pyridoxal‐5’‐phosphate (PLP), 300 mM NaCl). The cells were disrupted by ultrasonication in a continuous approach for 30 min at 4°C. Afterward, the cell debris was separated from the crude cell extract by centrifugation at 10°C and 14 000 rpm for 25 min using a Beckman Avanti JXN‐26 centrifuge (Beckman Coulter, Brea, USA). For protein purification, the Äkta Pure system (Cytiva, Marlborough, MA, USA) was used. Here, the crude cell extract was loaded on a 15 mL Ni‐NTA superflow column (Qiagen, Hilden, Germany) with a flow rate of 4 mL·min^1^. After loading the column, the unbound protein was removed from the column with 4 mL·min^−1^ equilibration buffer until the UV_250 nm_ signal decreased to the initial absorption. The unspecifically bound protein was washed with 50 mM KPi buffer (pH 7.5, 0.2 mM PLP, 300 mM NaCl) containing 25 mM imidazole, while the specifically bound *Cv*ATA was eluted afterwards with 300 mM imidazole (KPi buffer, pH 7.5, 0.2 mM PLP, 300 mM NaCl). After affinity chromatography, the protein fraction was desalted to remove imidazole and NaCl using a gel filtration method with 10 mM KPi buffer (pH 7.5, 0.2 mM PLP) as desalting buffer and a 1 L Sephadex G‐25 column (Cytiva, Marlborough, MA, USA). The purified and desalted *Cv*ATA solution was stored at −20°C overnight and freeze‐dried at −44°C and 0.16 mbar for 24 h. To control the success of the purification, SDS‐PAGE was performed for each purification fraction (Figure S2).

### 4.3 Photometric Scans of the Cofactor Pyridoxal‐5^′^‐Phosphate

The influence of different reaction compounds on the absorption spectrum of the cofactor PLP was investigated with a Shimadzu UV‐1800 spectrophotometer. Therefore, 0.1 mM PLP was dissolved in 100 mM phosphate buffer (pH 7.6) if not stated different. 1 mL of this solution was prepared in a quartz cuvette, and wavelength scans in a range between 250 and 500 nm were recorded in 2 nm increments and a total time span of 10 min. To detect the influence of metaraminol on the absorption spectrum of PLP, 500 µL of a 40 mM metaraminol bitartrate stock solution was added directly into the quartz glass cuvette containing 500 µL of a 0.2 mM PLP solution in 100 mM KPi buffer (pH 7.6). Directly after the addition of metaraminol, the wavelength scans were started and measured at 1 min intervals. The absorption spectra were analyzed and compared to the spectrum of a PLP solution without any additive.

### 4.4 Preparation and Detection of the Amination of (*R*)‐3‐OH‐PAC Toward Metaraminol

The amination toward metaraminol was investigated in this study under different conditions. Therefore, a 100 mM potassium phosphate (KPi) buffer (pH 7.5) was prepared containing 40 to 160 mM (*R*)‐3‐OH‐PAC, 250 mM to 1 M isopropylamine (6.25‐fold excess, pH adjusted to pH 7.5), and 1 mM PLP. As a biocatalyst, either 10 mg·mL^−1^ lyophilized whole cells harboring the *Cv*ATA or 10 mg·mL^−1^ lyophilized, purified *Cv*ATA were used, if not stated differently. In the case of purified *Cv*ATA, 10 mg·mL^−1^ lyophilized powder containing *Cv*ATA was used. According to protein quantification *via* the Bradford method [49], 60% of the lyophilizate was referred to *Cv*ATA, corresponding to 6 mg·mL^−1^. The reaction was conducted in open 1.5 mL glass vials at 30°C and 1000 rpm. 20 µL samples were taken at regular time spans of 1 h, after 20, and 24 h. The samples were diluted in 380 µL 50 % (v/v) acetonitrile in water to stop the reaction at the respective time points and centrifuged at 14 000 rpm for 3 min at room temperature. The (*R*)‐3‐OH‐PAC depletion and metaraminol formation were determined using the Thermo Scientific Ultimate 3000 UPLC system equipped with a UV–vis detector and a 250 × 4 mm, 5 μm LiChrospher 100 RP‐18 column (Merck KGaA, Darmstadt, Germany). The reaction compounds were eluted with a flow rate of 0.5 mL·min^−1^ using a gradient of 3% (v/v) acetonitrile in water with 0.1% (v/v) trifluoroacetic acid (TFA) to 100% water with 0.1% (v/v) TFA (1–18 min). Afterward, the initial concentration of 3% (v/v) acetonitrile with 0.1% (v/v) TFA was restored from 18 to 25 min. Under these conditions, PLP elutes after 5.9 min, metaraminol after 10.5 min, and (*R*)‐3‐OH‐PAC after 12.3 min. The analyte detection was performed at two wavelengths: *λ* = 254 nm and *λ* = 277 nm to control potential side product formation. The (*R*)‐3‐OH‐PAC and metaraminol concentrations were calculated according to the calibration curves depicted in Figures S3 and S4. All experiments were conducted in duplicates if not stated differently. The reaction yield was calculated based on the theoretical substrate concentration (Equation (1)):

(1) $$\text{yield}(t)=\frac{n_{Meta,t}}{n_{3\text{OHPAC},0}}$$

### 4.5 LC‐MS Analysis of Side‐Products

For the detection and identification of the product between metaraminol and PLP, samples containing 1 mM PLP and 1 mM metaraminol in 100 mM HEPES buffer (pH 7.6) were prepared and incubated at room temperature overnight. The samples were measured using a Thermo Scientific HPLC system equipped with a UV–vis detector measuring at a wavelength of *λ* = 250 nm. Prior to the measurement of each sample, the Atlantis T3 column (100 Å, 3 µm, 4.6 mm × 150 mm) (Waters Corporation, Milford, USA) was equilibrated with 0.6 mL·min^−1^ of 2% (v/v) acetonitrile in ultrapure water. 10 µL samples were injected, and their components were separated by applying a gradient of 2% (v/v) acetonitrile to 10% (v/v) acetonitrile in ultrapure water containing 0.1% (v/v) formic acid over 10 min at a flow rate of 0.6 mL·min^−1^. Afterward, the samples were fractionated in an ISQ EM mass spectrometer (Thermo Scientific, Waltham, USA) by electron spray ionization and measured in positive mode with a source voltage of 3000 V and an ion transfer temperature of 300°C. A mass range of 100–1000 Da was recorded, extracting the masses of 168, 248, 318, and 397 Da (Figures S9, S10, and S11).

For the detection of the possible side‐products of (*R*)‐3‐OH‐PAC, reaction batches were prepared according to the chapter above, and 20 µL samples were diluted 1:10 in 50% (v/v) acetonitrile in ultrapure water. The separation of the reaction compounds was also conducted according to the HPLC method described in the chapter above. After separation, the sample compounds were fractionated in a Sciex 6600QTOF (Sciex, Toronto, Canada) equipped with a DuoSpray source at a temperature of 450°C and an ion spray voltage of 5500 V. A mass range of 50–1000 Da was recorded (Figures S15 and S16).

### 4.6 Quantum Mechanics Computations of the Absorption Spectrum of the Pictet–Spengler and Oxazolidine Product From Metaraminol and PLP

A multistep quantum chemistry workflow was employed to predict UV–vis spectra for four chemical products (PLP, the Pictet–Spengler product, the oxazolidine product, and the Schiff base) (Figure 2B). The protonation states for PLP and the Schiff base were taken from [50, 51, 52, 53], and the cyclic secondary amine in the Pictet–Spengler product was considered protonated [54]. The protonation states and the stereoisomers selected for the different chemical moieties are shown in Figure 2B. Other protonation states and regio‐stereoisomers were sampled, and the best‐fitting protonation state/regio‐stereoisomer to the experimental spectra was selected. A total of two protonation states for PLP, two protonation states for the Schiff base, three protonation states and two regio‐stereoisomers for *ortho‐* and *para‐*Pictet–Spengler product, respectively, and two protonation states and eight regio‐stereoisomers for the oxazolidine product were calculated (Figure S13). The summary of all the structures used in this study is provided in Table S1.

For each compound, the geometry was initially optimized using GFN2‐xTB, a self‐consistent tight‐binding quantum chemical method [55], with the analytical linearized Poisson–Boltzmann model for solvation in water to generate starting structures [56]. Conformer searches were then conducted with CREST [57] to identify the lowest‐energy conformers. These conformers underwent further optimization at the r2SCAN‐3c density functional level [58] with the CPCM solvation model [59], as implemented in ORCA 5.0.3 [60, 61]. The r2SCAN‐3c method employs a compact triple‐zeta basis set with integrated dispersion and geometrical counterpoise corrections for efficient and accurate geometry optimization [58]. UV–Vis absorption spectra were subsequently calculated in ORCA with the CAM‐B3LYP functional [62] using the def2‐SVP basis set with the CPCM solvation model. Excited states were computed using the simplified time‐dependent density functional theory approach (sTD‐DFT) [63], which allows fast computation of electronic UV spectra of molecules with 500–1000 atoms. sTD‐DFT in combination with CAM‐B3LYP has been shown to be the most accurate combination in independent benchmark calculations of porphyrinoids and phtalocyanines [64, 65]. All excitation up to 10 eV were considered and processed using the orca_mapspc utility [61].

### 4.7 Continuous In Situ Extraction of Metaraminol

A continuous in situ extraction setup was established to increase the yield of metaraminol and operated over several days. Two 20 mL glass vials with screw caps were connected via flexible tubing to enable forward and back extraction. Each vial was charged with 7 mL of aqueous phase. The reaction phase consisted of 100 mM KPi buffer at pH 8.5, to which 250 mM L‐alanine and 0.1 mM PLP were added. The back‐extraction phase was prepared as an acidic solution at pH 3.0 with 250 mM HCl. The organic solvent (1‐octanol) was transferred between the glass vials using two KNF SIMDOS membrane dosing pumps at a pumping rate of 2 mL·min^−1^. At the start of each experiment, the desired concentration of purified enzyme (0.5, 2, 10 mg·mL^−1^) was added to the reaction‐phase vial. The organic phase consisted initially of 20 mL of 1‐octanol saturated with 30 mM (*R*)‐3‐OH‐PAC. To feed the main substrate ((*R*)‐3‐OH‐PAC) into the system, the organic phase was transferred to the back‐extraction vial, and the pumps were activated. Due to the distribution equilibrium of (*R*)‐3‐OH‐PAC, it partitioned between both phases and was successively converted to metaraminol in the reaction phase. Each vial was maintained at 30°C using heating plates and stirred by magnetic stirrers. The stirring speed was adjusted to generate a stable vortex while preventing significant dispersion of phases, avoiding excessive enzyme contact at the interface. Each experiment lasted 70 h and was performed in duplicate. A photograph of the experimental setup can be found in the supporting information (Figure S5).

Samples were withdrawn from the back‐extraction phase throughout the reaction and analyzed by HPLC for metaraminol concentration according to the published quantification method of metaraminol in pure substance extraction systems from Mack et al. [11]. The absolute amount of metaraminol was calculated from these concentration measurements, the sample weights, and the total mass of the back‐extraction phase. Yield determinations were based on the initial mass of (*R*)‐3‐OH‐PAC in the organic phase (Equation (2)):

(2) $$\text{yield}(t)=\frac{c_{Meta}\left(t\right)\cdot V_{BEx}\left(t\right)+\sum_{i}^{n_{\text{samples}}}c_{Meta_{i}}\cdot V_{i}}{c_{3OHPAC_{0}}\cdot V_{\text{reaction}}}$$

## Supporting Information

Additional supporting information can be found online in the Supporting Information section. SDS‐PAGE gels, further experiments on reaction engineering, details on quantum mechanics computations and LC‐MS data are given in the Supporting Information. **Supporting** **Fig.** **S1:** SDS‐PAGE of whole cells and crude cell extract from amine transaminase *Cv*ATA after cultivation. **Lane 1**: 4 µL prestained protein ladder marker, **lane 2**: 7 µL soluble protein fraction, **lane 3**: 7 µL insoluble protein fraction, **lane 4**: 7 µL crude cell extract, **lane 5**: 10 µL prestained protein ladder marker. **Supporting Fig. S2:** SDS‐PAGE of *Cv*ATA samples after IMAC. IMAC was performed twice and afterward the desalting fractions were pooled. *Cv*ATA monomer has a molecular weight of ∼54 kDa highlighted with the red box. CCE: crude cell extract; F: flow through after loading the column; W: washing fraction of unspecific bound protein; Elu: Elution fraction containing 300 mM imidazole; DS: desalting fraction. **Supporting Fig. S3:** Calibration curve of the substrate (*R*)‐3‐OH‐PAC ranging from 0 mM to 5 mM measured via an UV–vis detector at λ = 254 nm. The resulting linear equation was used to calculate the substrate concentration in the unknown samples. **Supporting Fig. S4:** Calibration curve of the target product metaraminol ranging from 0 mM to 5 mM measured via an UV–vis detector at λ = 254 nm. The resulting linear equation was used to calculate the product concentration in the unknown samples. **Supporting Fig. S5** **:** Experimental setup of the continuous in situ extraction of metaraminol comprising the aqueous reaction system (pH 8.5) and the back extraction phase (pH 3.0) in the glass vials. The organic extraction phase (20 mL 1‐octanol), containing 30 mM (*R*)‐3‐OH‐PAC, is continuously pumped between both vials. **Supporting Fig. S6** **:** Relative initial product formation rate of CvATA after the incubation for 3 h in various (*R*)‐3‐OH‐PAC concentrations ranging from 40 mM to 160 mM. Prior to the reaction, 10 mg·mL^‐1^ lyophilized whole cell *Cv*ATA was incubated in 1 mL 100 mM KPi buffer (pH 7.5) containing 40‐160 mM (*R*)‐3‐OH‐PAC and 1 mM PLP. After 3 h of incubation, the incubation mixture was centrifuged and 1 mL 100 mM KPi buffer (pH 7.5) was added to the cells containing 20 mM (*R*)‐3‐OH‐PAC, 125 mM IPA and 1 mM PLP. T = 30°C, V = 1 mL, t = 24 h. n = 2, mean ± SD. **Supporting Fig. S7** **:** Metaraminol production upon feeding crude cell extract containing *Cv*ATA, the amine donor IPA and the substrate (*R*)‐3‐OH‐PAC under reduced pressure. Reaction conditions: 40 mM (*R*)‐3‐OH‐PAC, 250 mM IPA, 1 mM PLP and 15 mg/mL crude cell extract containing *Cv*ATA in 100 mM KPi buffer (pH 7.6). T = 30°C, p = 750 mbar, 15 mg/mL crude cell extract, 250 mM IPA and 40 mM (*R*)‐3‐OH‐PAC were fed after 4 h. n = 1. **Supporting Fig. S8** **:** (A) Absorption spectra of 0.1 mM PLP in 100 mM KPi buffer (pH7.6) measured every minute over a time span of 10 min. (B) Absorption spectra of 0.1 mM PLP in 100 mM KPi buffer (pH7.6) in the presence of 20 mM (1S,2R)‐norephedrine measured every minute over a time span of 10 min. **Supporting Fig. S9** **:** LC‐MS results of 1 mM pyridoxal‐5’‐phosphate (PLP) in 100 mM HEPES buffer (pH 7.6) (A) Chromatogram of PLP with a retention time of 1.75 min. (B) mass spectrum of PLP with extracted m/z of 248.1, 266.2, 495.1. **Supporting Fig. S10** **:** LC‐MS results of 1 mM metaraminol in 100 mM HEPES buffer (pH 7.6) (A) Chromatogram of metaraminol with a retention time of 1.93 min. (B) mass spectrum of metaraminol with extracted m/z of 150.2 and 168.2. **Supporting Fig. S11** **:** LC‐MS results of 1 mM PLP and 1 mM metaraminol in 100 mM HEPES buffer in one batch (pH 7.6) (A) Chromatogram of the reaction mixture with compounds exhibiting retention times of 1.77 min, 1.94 min, 2.26 min, 2.66 min (metaraminol) and 3.77 min . (B) Mass spectra of each extracted peak showing the same m/z of 397.2. With a peak at 2.66 min, an m/z of 168.2 was extracted being most likely the metaraminol. **Supporting Fig. S12** **:** Probability density distribution of the attack angle observed in the CREST ensemble of the Schiff base (Schiff2). The typical ortho‐attack angle and para‐attack angle distributions for the Pictet–Spengler product formation are shown as dark red and pink histograms, respectively. The typical attack angle for the oxazolidine derivative is shown as blue histograms. The ideal Bürgi–Dunitz angle is shown as a dotted gold vertical line. A) Attack angle comparison between Oxazolidine‐like (blue) and o‐Pictet–Spengler (red). B) Attack angle comparison between Oxazolidine‐like (blue) and p‐Pictet–Spengler (pink). C) Attack angle comparison between o‐Pictet–Spengler (red) and p‐Pictet–Spengler (pink). **Supporting Fig. S13** **:** Experimental (top) and computed UV–vis spectra identify the Pictet–Spengler and the oxazolidine derivatives. The computed spectra are reported for the PLP (in green, two protonation states selected, with PLP1 better fitting to the experimental spectrum; PLP1 protonation was also in agreement with Limbach et al., Sharif et al. and Chan‐Huot et al. [51, 52, 53]. Schiff base spectrum is shown in orange (two protonation states selected, with Schiff2 better fitting to the experimental spectrum). Pictet–Spengler spectra are shown for the ortho‐derivative (in brown, three protonation states, two regio‐stereoisomers, with o‐PS_3_1 better fitting to experimental spectrum) and the para‐derivative (in purple, three protonation, two regio‐stereoisomers, with p‐PS_3_1 better fitting to experimental spectrum). The oxazolidine derivative spectrum is shown for two protonation states (in blue, eight regio‐stereoisomer combinations per protonation state, with Oxa_1_6 better fitting to the experimental spectrum). **Supporting** **Fig** **S14:** PLP feed during the reductive amination: (*R*)‐3‐OH‐PAC depletion and metaraminol production. Reaction conditions: 0.6 mg·mL^−1^ purified *Cv*ATA (concentration determined via the Bradford method), 20 mM (*R*)‐3‐OH‐PAC, 125 mM isopropylamine, 0.1 mM PLP in 100 mM KPi buffer (pH 7.6). V = 1 mL, T = 30°C, 1000 rpm. After 2.5 h, 4.5 h and 5.5 h additional 0.1 mM PLP was fed. n = 1. **Supporting Fig. S15:** (A) LC chromatogram of the reaction mixture containing 40 mM (*R*)‐3‐OH‐PAC and 250 mM IPA with 1 mM PLP with 20 mg·mL^­1^ whole cell *Cv*ATA. Metaraminol elutes after 10.2 min, (*R*)‐3‐OH‐PAC elutes after 12.1 min. (B) Mass spectrum of the unknown peak at 11.1 min, assumed to be 1‐(3‐hydroxyphenyl)propane‐1,2‐dione, the oxidation product of (*R*)‐3‐OH‐PAC. **Supporting Fig. S16:** (A) LC chromatogram of the reaction mixture containing 40 mM (*R*)‐3‐OH‐PAC and 250 mM IPA with 1 mM PLP with 20 mg·mL^−1^ whole cell *Cv*ATA. Metaraminol elutes after 10.2 min, (*R*)‐3‐OH‐PAC elutes after 12.1 min. The to be analyzed compound elutes after 12.7 min. (B) Mass spectrum of the unknown peak at 12.7 min, assumed to be the imine of (*R*)‐3‐OH‐PAC and IPA. **Supporting** **Table** **S1:** Protonation states and regio‐stereoisomers considered in this study. The green‐labeled structures are the ones shown in Figure 2B.

## Conflicts of Interest

The authors declare no conflicts of interest.

## Supporting information

Supplementary Material

## Data Availability

The data that support the findings of this study are available from the corresponding author upon reasonable request.
