# Supplementary material for: An Integrated Engineering Approach to Intensify the Biocatalytic Metaraminol Synthesis
Source: ChemSusChem. 2026 Feb 13;19(4):e202502108. doi: 10.1002/cssc.202502108 (PMC12904732; doi:10.1002/cssc.202502108)
Supplement: Supplementary file 1 — Supplementary Material [file CSSC-19-e202502108-s001.pdf]

Supporting Information

Gene sequence of amine transaminase of *Chromobacterium violaceum* (*Cv*ATA) 2
Amino acid sequence of amine transaminase of *Chromobacterium violaceum* (*Cv*ATA) 2
Sodium dodecyl sulfate polyacrylamide gel electrophoresis (SDS-PAGE) 2
Expression control of *Cv*ATA after cultivation via SDS-PAGE 2
SDS-PAGE after IMAC of *Cv*ATA as purification control 3
Calibration curves of the substrate (*R*)-3-OH-PAC and the target product metaraminol 4
Experimental setup of the continuous *in situ* extraction of metaraminol 6
Reaction engineering strategies for metaraminol synthesis using IPA as amine donor 6
Substrate inactivation tested with various (*R*)-3-OH-PAC concentrations 6
Reaction performed under reduced pressure: Substrate and biocatalyst feed 7
Side-product formation: Complex formation of pyridoxal-5'-phosphate and metaraminol 8
Absorption spectra of pyridoxal-5'-phosphate (PLP) and norephedrine 8
LC-MS results 8
LC-MS data from 1 mM pyridoxal-5'-phosphate in 100 mM HEPES buffer 9
LC-MS data from 1 mM metaraminol bitartrate in 100 mM HEPES buffer 10
LC-MS data from 1 mM PLP and 1 metaraminol in 100 mM HEPES buffer 10
Quantum mechanics computations of the absorption spectrum of the Pictet-Spengler and oxazolidine product from metaraminol and PLP 13
PLP-feed during the *Cv*ATA catalyzed reductive amination of (*R*)-3-OH-PAC towards metaraminol 20
Side-product formation of the substrate (*R*)-3-OH-PAC 21

Gene sequence of amine transaminase of *Chromobacterium violaceum* (*Cv*ATA)

CATCATCATCATCATCATATGCAGAAGCAACGTACGACCAGCCAATGGCGCGAACTGGATGCCGCCCATCACCTGCATCCGT
TCACCGATACCGCATCGCTGAACCAGGCGGGCGCGCGCGTGATGACGCGCGGAGAGGGCGTCTACCTGTGGGATTCGGAAGG
CAACAAGATCATCGACGGCATGGCCGGACTGTGGTGCGTGAACGTCGGCTACGGCCGCAAGGACTTTGCCGAAGCGGCGCGC
CGGCAGATGGAAGAGCTGCCGTTCTACAACACCTTCTTCAAGACCACCCATCCGGCGGTGGTCGAGCTGTCCAGCCTGCTGG
CTGAAGTGACGCCGGCCGGTTTCGACCGCGTGTTCTATACCAATTCCGGTTCCGAATCGGTGGACACCATGATCCGCATGGT
GCGCCGCTACTGGGACGTGCAGGGCAAGCCGGAGAAGAAGACGCTGATCGGCCGCTGGAACGGCTATCACGGCTCCACCATC
GGCGGCGCCAGCCTGGGCGGCATGAAGTACATGCACGAGCAGGGCGACTTGCCGATTCCGGGCATGGCCCACATCGAGCAGC
CTTGGTGGTACAAGCACGGCAAGGACATGACGCCGGACGAGTTCGGCGTGGTGGCCGCGCGCTGGCTGGAAGAGAAGATTCT
GGAAATCGGCGCCGACAAGGTGGCCGCCTTCGTCGGCGAACCCATCCAGGGCGCCGGCGGCGTGATCGTCCCGCCGGCCACC
TACTGGCCGGAAATCGAGCGCATTTGCCGCAAGTACGACGTGCTGCTGGTGGCCGACGAAGTGATCTGCGGCTTCGGGCGTA
CCGGCGAATGGTTCGGCCATCAGCATTTCGGCTTCCAGCCCGACCTGTTCACCGCCGCCAAGGGCCTGTCCTCCGGCTATCT
GCCGATAGGCGCGGTCTTTGTCGGCAAGCGCGTGGCCGAAGGCCTGATCGCCGGCGGCGACTTCAACCACGGCTTCACCTAC
TCCGGCCACCCGGTCTGCGCCGCCGTCGCCCACGCCAACGTGGCGGCGCTGCGCGACGAGGGCATCGTCCAGCGCGTCAAGG
ACGACATCGGCCCGTACATGCAAAAGCGCTGGCGTGAAACCTTCAGCCGTTTCGAGCATGTGGACGACGTGCGCGGCGTCGG
CATGGTGCAGGCGTTCACCCTGGTGAAGAACAAGGCGAAGCGCGAGCTGTTCCCCGATTTCGGCGAGATCGGCACGCTGTGC
CGCGACATCTTCTTCCGCAACAACCTGATCATGCGGGCATGCGGCGACCACATCGTGTCGGCGCCGCCGCTGGTGATGACGC
GGGCGGAAGTGGACGAGATGCTGGCGGTGGCGGAACGCTGTCTGGAGGAATTCGAGCAGACGCTGAAGGCGCGCGGGCTGGC
TTAG

Amino acid sequence of amine transaminase of *Chromobacterium violaceum* (*Cv*ATA)

HHHHHHMQKQRTTSQWRELDAAHHLHPFTDTASLNQAGARVMTRGEGVYLWDSEGNKIIDGMAGLWCVNVGYGRKDFAEAAR
RQMEELPFYNTFFKTTHPAVVELSSLLAEVTPAGFDRVFYTNSGSESVDTMIRMVRRYWDVQGKPEKKTLIGRWNGYHGSTI
GGASLGGMKYMHEQGDLPIPGMAHIEQPWWYKHGKDMTPDEFGVVAARWLEEKILEIGADKVAAFVGEPIQGAGGVIVPPAT
YWPEIERICRKYDVLLVADEVICGFGRTGEWFGHQHFGFQPDLFTAAKGLSSGYLPIGAVFVGKRVAEGLIAGGDFNHGFTY
SGHPVCAAVAHANVAALRDEGIVQRVKDDIGPYMQKRWRETFSRFEHVDDVRGVGMVQAFTLVKNKAKRELFPDFGEIGTLC
RDIFFRNNLIMRACGDHIVSAPPLVMTRAEVDEMLAVAERCLEEFEQTLKARGLA

Sodium dodecyl sulfate polyacrylamide gel electrophoresis (SDS-PAGE)

Expression control of *Cv*ATA after cultivation via SDS-PAGE

After cultivation, 1 mL samples were drawn from each shake flask and the optical density at λ = 600 nm was adjusted to 0.45. The samples were centrifuged with a bench top centrifuge for 3 min at 14 000 rpm. The supernatant was discarded while the cell pellets were stored at -20 °C for at least one night. The cell pellets were thawed and resuspended in 50 µL lysis buffer (1 mg·mL^{-1} lysozyme, 0.5 µL·mL^{-1} benzonase, and 0.5 mg·mL^{-1} polymyxin B in 100 mM KPi buffer, pH 7.5). The samples were incubated for 20 min on ice and centrifuged in a benchtop centrifuge for 25 min at 14 000 rpm and 10 °C. The supernatant was transferred in a fresh 1.5 mL reaction tube and is referred to as soluble protein fraction. The pellet was resuspended in 50 µL 7 M urea solution and centrifuged for 40 min at 14000 rpm and 10 °C. The supernatant was again transferred in a fresh 1.5 mL reaction tube and is referred to as insoluble protein fraction. 3 µL NuPAGE reducing agent (Invitrogen, Thermo Scientific) and 7.5 µL NuPAGE SDS sample buffer (Invitrogen, Thermo Scientific) were added to 19.5 µL of each protein sample. 7 µL of each prepared sample was loaded on a NuPAGE 4-12 % Bis-Tris gel and

electrophoresis was performed in MES-buffer at 200 V, 0.1 A and 15 W for 60 min. The gel was stained with Coomassie Brilliant Blue for 60 min and destained with ultrapure water overnight.

In Figure 1, the SDS-PAGE after cultivation is shown. Both protein fractions, soluble (lane 2) and insoluble (lane 3), different sample amounts show an overexpression of the *Cv*ATA (molecular mass of a monomer: ~54 kDa). Soluble enzyme is expressed approximately in the same amount as insoluble proteins (after urea treatment). The band at approximately 14 kDa is the lysozyme. In the crude cell extract further cell proteins are detectable and the enzyme band is stronger compared to whole cells.

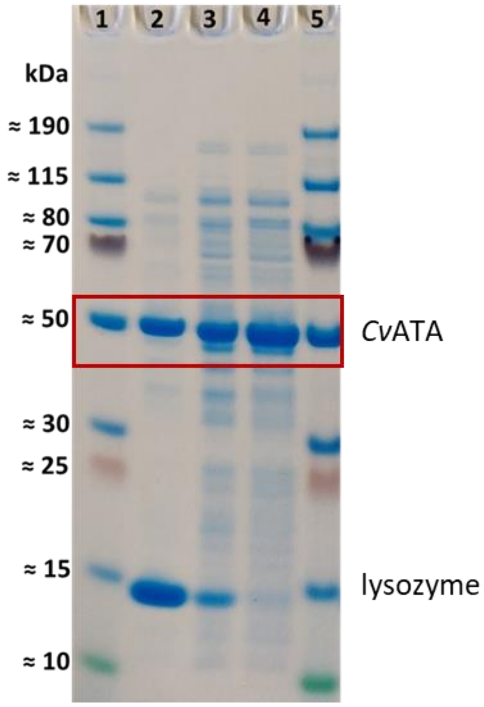

Figure 1: SDS-PAGE of whole cells and crude cell extract from amine transaminase *Cv*ATA after cultivation. **Lane 1**: 4 µL prestained protein ladder marker, **lane 2**: 7 µL soluble protein fraction, **lane 3**: 7 µL insoluble protein fraction, **lane 4**: 7 µL crude cell extract, lane 5: 10 µL prestained protein ladder marker.

SDS-PAGE after IMAC of *Cv*ATA as purification control

After purification of *Cv*ATA, protein concentration of each fraction (crude cell extract, flow through, washing step, elution, desalting) was determined in technical triplicates according to Bradford *et al.*[49] and calculated according to an *a priori* performed calibration curve with BSA as reference protein. The protein end concentration in the SDS samples was adjusted to 1.5 mg·mL^{-1}. 19.5 µL of the samples, from the cultivation and purification, were added to 7.5 µL Invitrogen SDS buffer (Thermo Scientific, Waltham, USA) and 3 µL NuPAGE Invitrogen reducing agent (Thermo Scientific, Waltham, USA) and were boiled for 5 min at 95 °C. 10 µL of the prepared samples were loaded per lane of a NuPAGE 4-16 % Bis-Tris gel (Thermo Scientific, Waltham, USA).

The SDS-PAGE of the samples of each purification step are depicted in Figure 2.

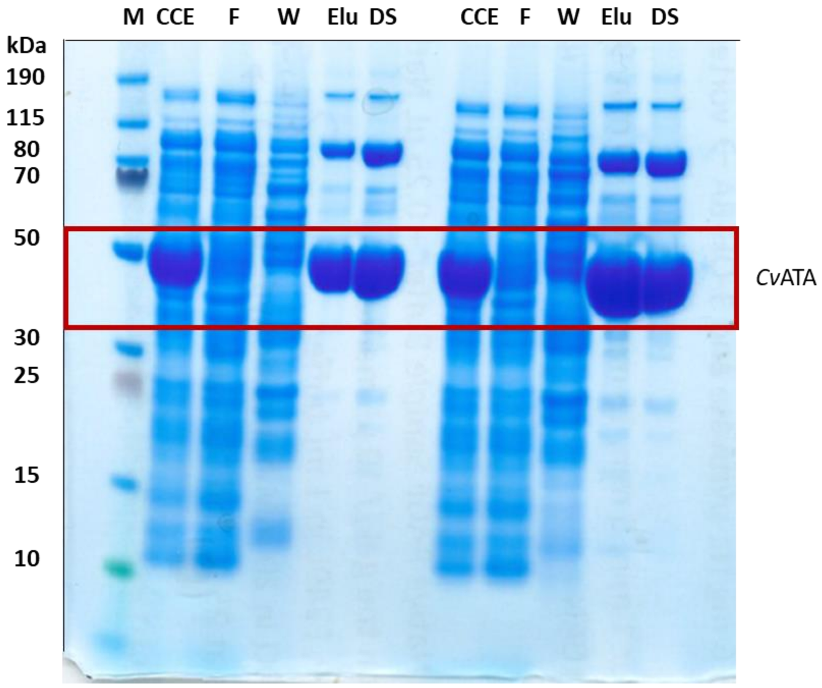

Figure 2: SDS-PAGE of *Cv*ATA samples after immobilized metal affinity chromatography (IMAC). IMAC was performed twice and afterward the desalting fractions were pooled. *Cv*ATA monomer has a molecular weight of ~54 kDa highlighted with the red box. CCE: crude cell extract; F: flow through after loading the column; W: washing fraction of unspecific bound protein; Elu: Elution fraction containing 300 mM imidazole; DS: desalting fraction.

Calibration curves of the substrate (*R*)-3-OH-PAC and the target product metaraminol

A 10 mM stock solution of (*R*)-3-OH-PAC in 100 mM KPi buffer (pH 7.6) was prepared and diluted with KPi buffer to reach the respective concentration between 0 mM and 10 mM. Afterward, the samples were diluted 1:2 with acetonitrile (Figure 3). The samples were measured with the HPLC method described in the Material and Methods section.

For the calibration curve of metaraminol bitartrate, the calibration was prepared similarly to the calibration standards from (*R*)-3-OH-PAC stated above.

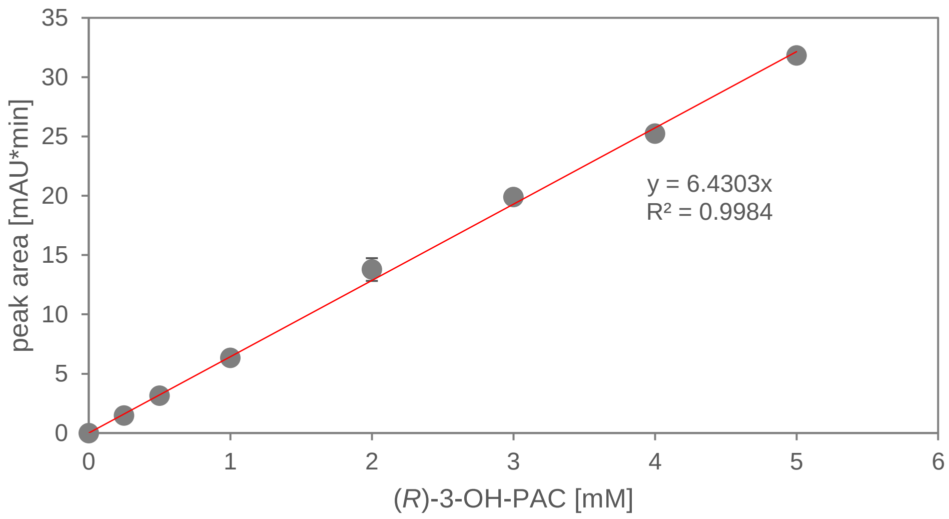

Figure 3: Calibration curve of the substrate (*R*)-3-OH-PAC ranging from 0 mM to 5 mM measured via an UV-Vis detector at λ = 254 nm. The resulting linear equation was used to calculate the substrate concentration in the unknown samples.

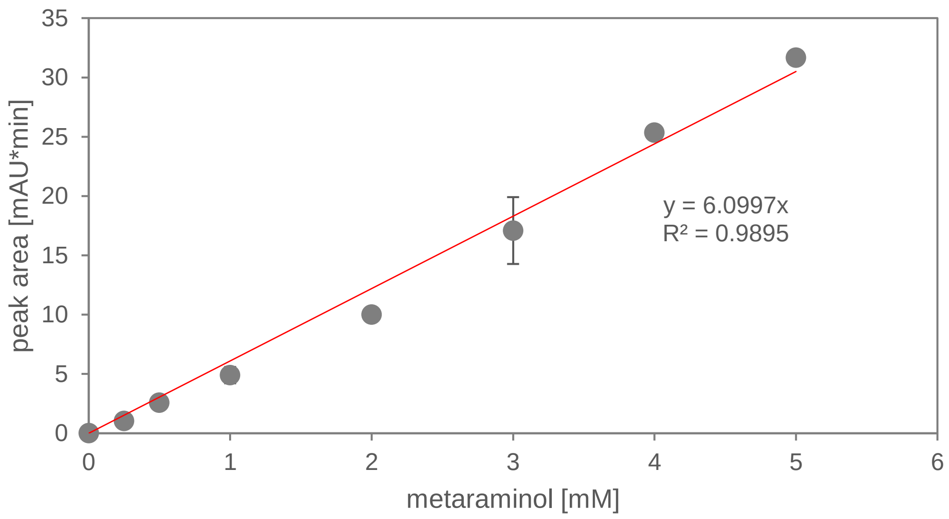

Figure 4: Calibration curve of the target product metaraminol ranging from 0 mM to 5 mM measured via an UV-Vis detector at λ = 254 nm. The resulting linear equation was used to calculate the product concentration in the unknown samples.

Experimental setup of the continuous *in situ* extraction of metaraminol

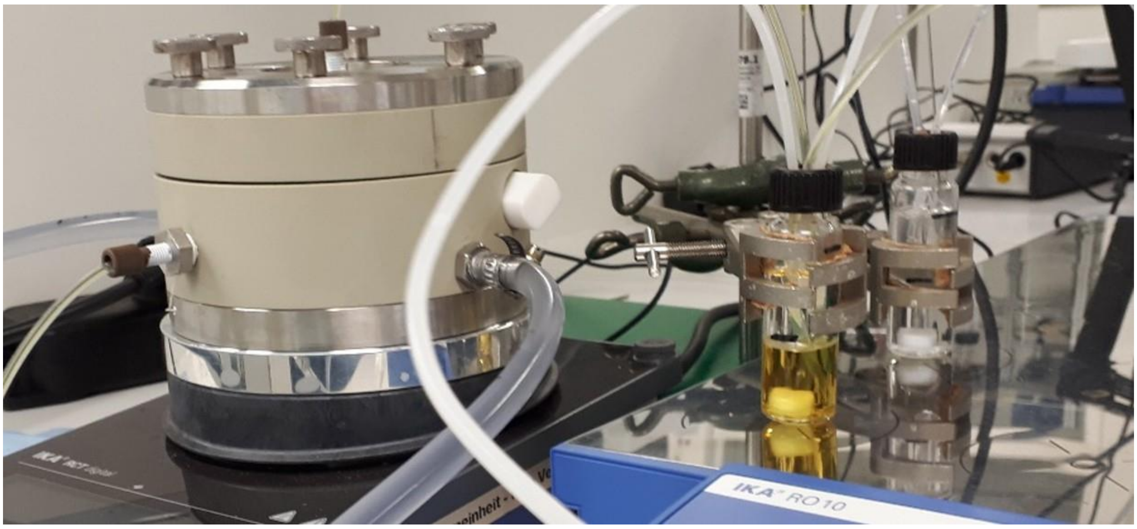

Figure 5: Experimental setup of the continuous *in situ* extraction of metaraminol comprising the aqueous reaction system (pH 8.5) and the back extraction phase (pH 3.0) in the glass vials. The organic extraction phase (20 mL 1-octanol), containing 30 mM (*R*)-3-OH-PAC, is continuously pumped between both vials.

Reaction engineering strategies for metaraminol synthesis using IPA as amine donor

Substrate inactivation tested with various (*R*)-3-OH-PAC concentrations

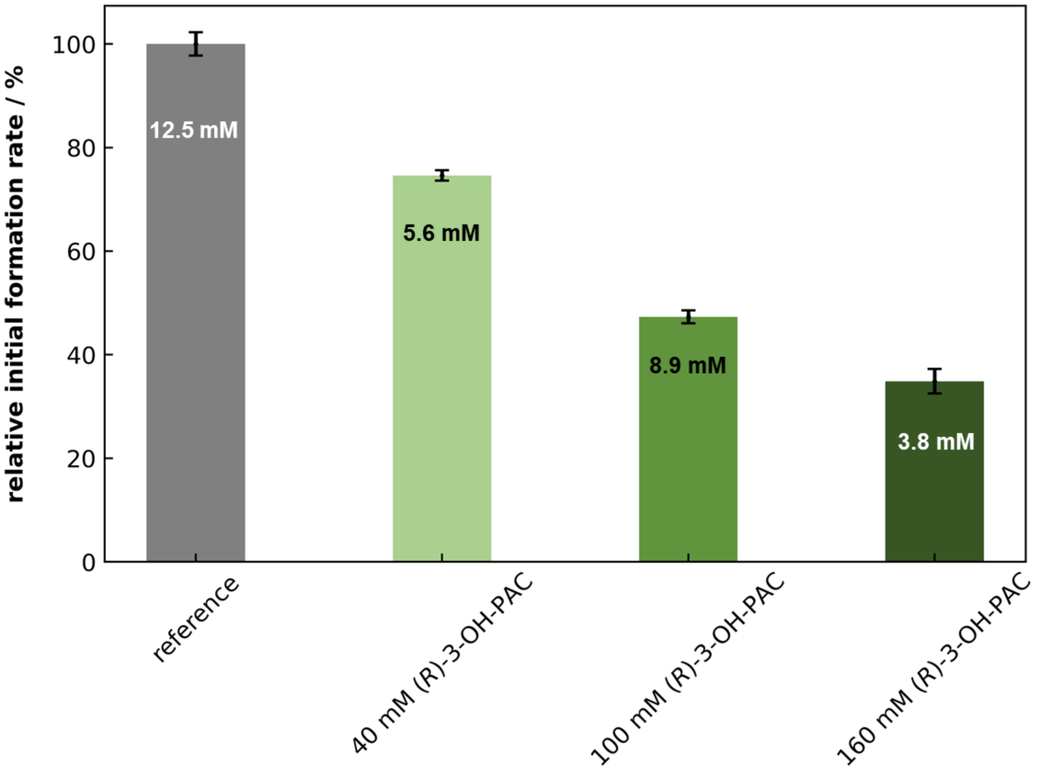

Figure 6: Relative initial product formation rate of *Cv*ATA after the incubation for 3 h in various (R)-3-OH-PAC concentrations ranging from 40 mM to 160 mM. Prior to the reaction, 10 mg·mL-1 lyophilized whole cell *Cv*ATA was incubated in 1 mL 100 mM KPi buffer (pH 7.5) containing 40-160 mM (*R*)-3-OH-PAC and 1 mM PLP. After 3 h of incubation, the incubation mixture was centrifuged and 1 mL 100 mM KPi buffer (pH 7.5) was added to the cells containing 20 mM (*R*)-3-OH-PAC, 125 mM IPA and 1 mM PLP. T = 30 °C, V = 1 mL, t = 24 h. n = 2, mean ± SD.

Reaction performed under reduced pressure: Substrate and biocatalyst feed

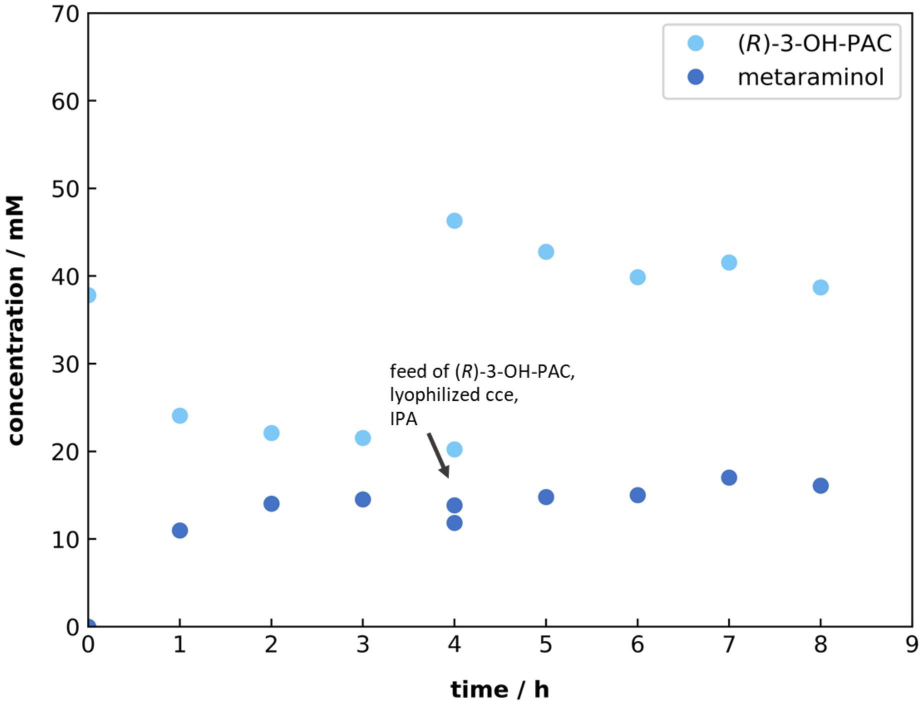

Figure 7: Metaraminol production upon feeding crude cell extract containing *Cv*ATA, the amine donor IPA and the substrate (*R*)-3-OH-PAC under reduced pressure. Reaction conditions: 40 mM (*R*)-3-OH-PAC, 250 mM IPA, 1 mM PLP and 15 mg/mL crude cell extract containing *Cv*ATA in 100 mM KPi buffer (pH 7.6). T = 30 °C, p = 750 mbar, 15 mg/mL crude cell extract, 250 mM IPA and 40 mM (*R*)-3-OH-PAC were fed after 4 h. n = 1.

Side-product formation: Complex formation of pyridoxal-5'-phosphate and metaraminol

Absorption spectra of pyridoxal-5'-phosphate (PLP) and norephedrine

Absorption spectra of PLP were determined as blank in 100 mM KPi buffer (pH 7.6) without the addition of another substance (Figure 8, A). In Figure 8, B the absorption spectra of 0.1 mM PLP are shown in the presence of 20 mM (1*S*,2*R*)-norephedrine. The experiments were performed according to the description in the Experimental section.

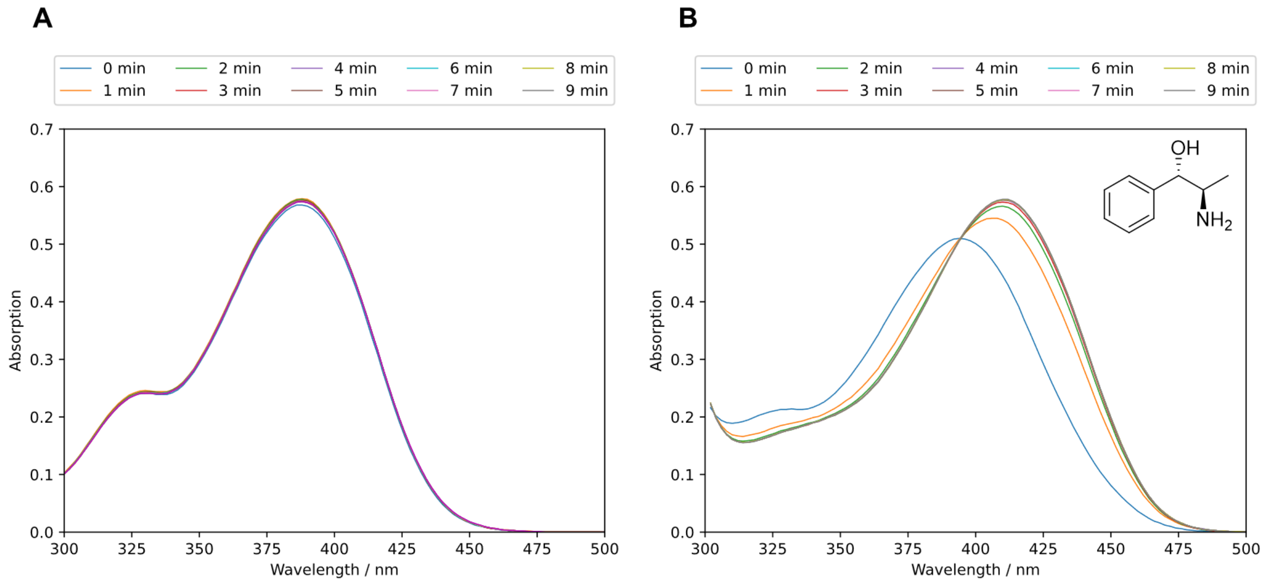

Figure 8: (A) Absorption spectra of 0.1 mM PLP in 100 mM KPi buffer (pH7.6) measured every minute over a time span of 10 min. (B) Absorption spectra of 0.1 mM PLP in 100 mM KPi buffer (pH7.6) in the presence of 20 mM (1*S*,2*R*)-norephedrine measured every minute over a time span of 10 min.

Contrary to the absorption spectra of PLP in the presence of 20 mM metaraminol, the absorption spectra of PLP in the presence of (1*S*,2*R*)-norephedrine do not show the formation of a new absorption maximum at 325 nm. However, after 1 min, the absorption maximum shifts from initially ~390 nm to 410 nm in the presence of (1*S*,2*R*)-norephedrine potentially indicating the formation of a Schiff base between PLP and the amine group of (1*S*,2*R*)-norephedrine. Thus, the hydroxy group in *meta*-position as a substituent of the aromatic group of metaraminol seems to have a strong influence on the complex formation with PLP.

LC-MS results

In the following sections, the LC-MS results of the complex formation between PLP and metaraminol are shown. First, the pure components were measured and afterward the reaction mixture with both, metaraminol and PLP in one pot.

LC-MS data from 1 mM pyridoxal-5'-phosphate in 100 mM HEPES buffer

A

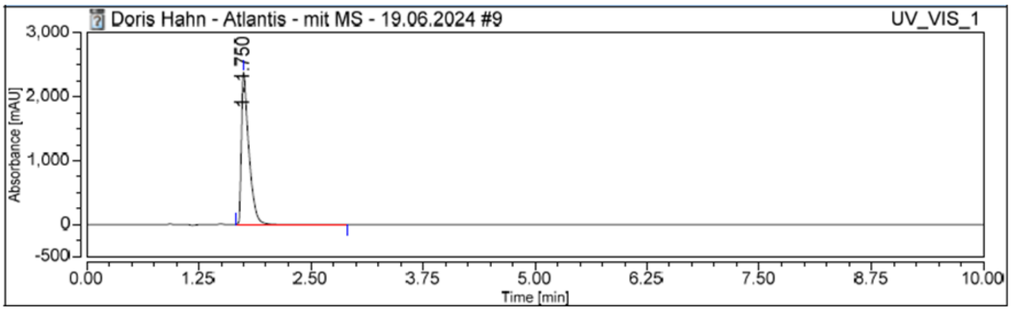

B

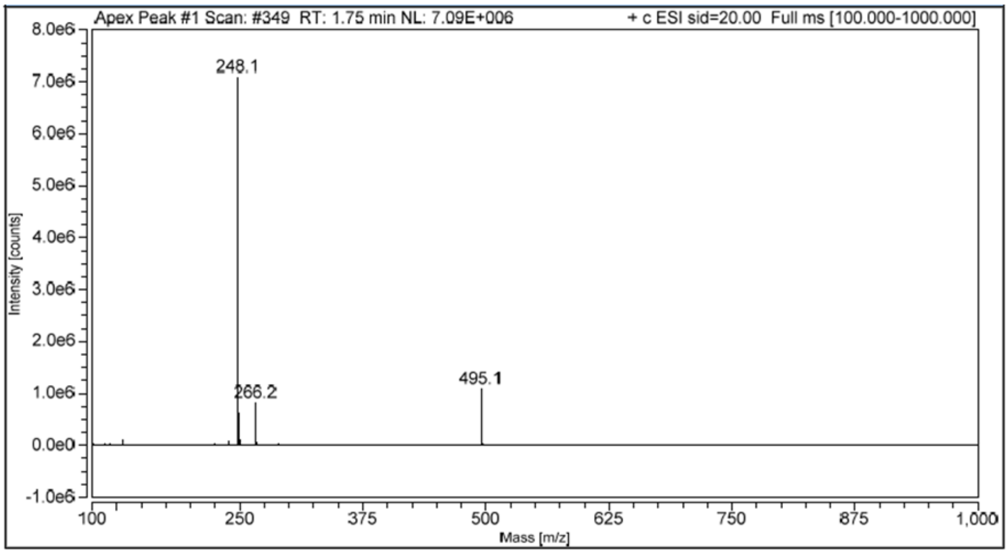

Figure 9: LC-MS results of 1 mM pyridoxal-5'-phosphate (PLP) in 100 mM HEPES buffer (pH 7.6) (A) Chromatogram of PLP with a retention time of 1.75 min. (B) mass spectrum of PLP with extracted m/z of 248.1, 266.2, 495.1.

LC-MS data from 1 mM metaraminol bitartrate in 100 mM HEPES buffer

A

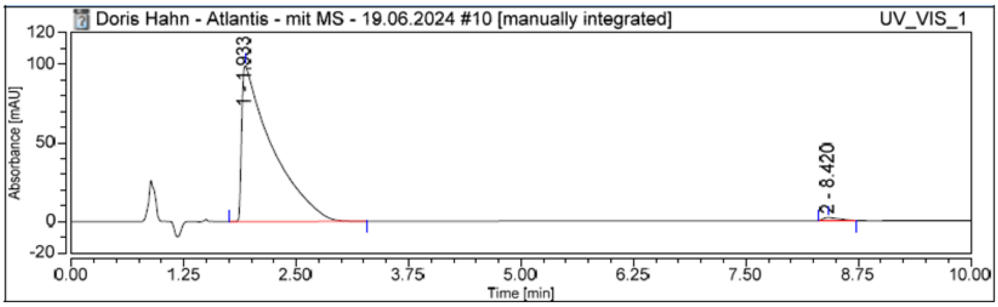

B

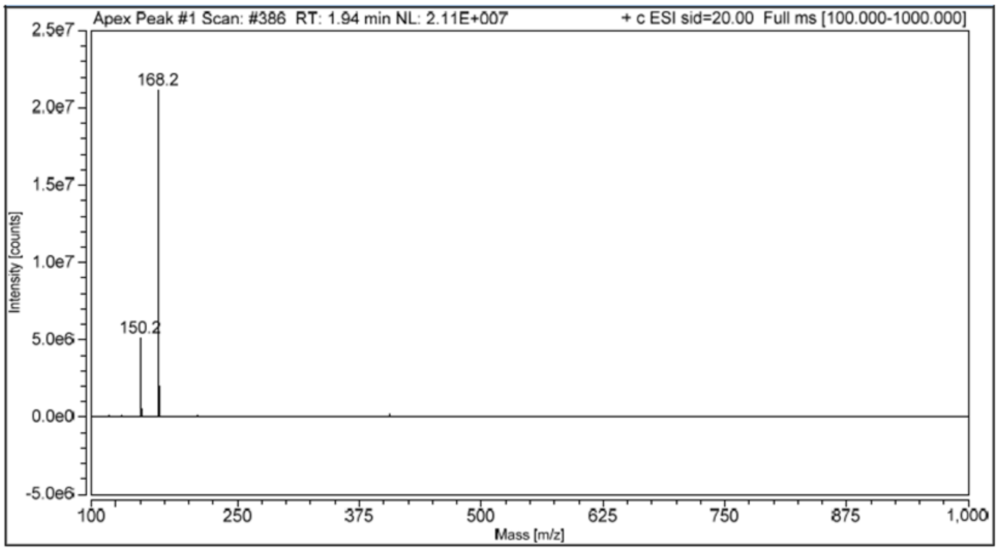

Figure 10: LC-MS results of 1 mM metaraminol in 100 mM HEPES buffer (pH 7.6) (A) Chromatogram of metaraminol with a retention time of 1.93 min. (B) mass spectrum of metaraminol with extracted m/z of 150.2 and 168.2.

LC-MS data from 1 mM PLP and 1 metaraminol in 100 mM HEPES buffer

A

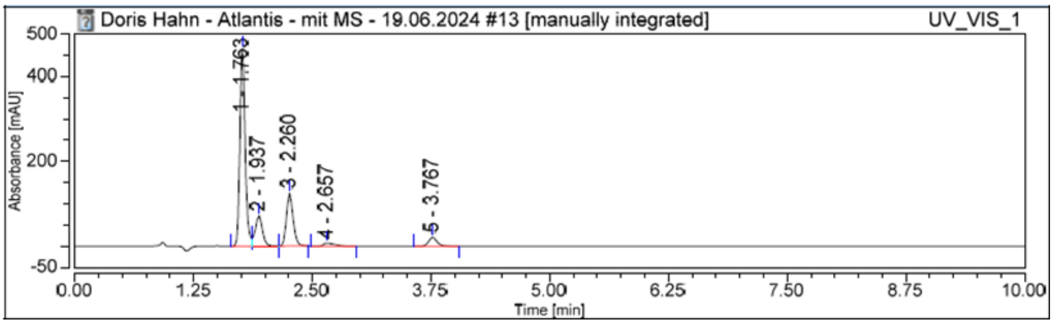

B

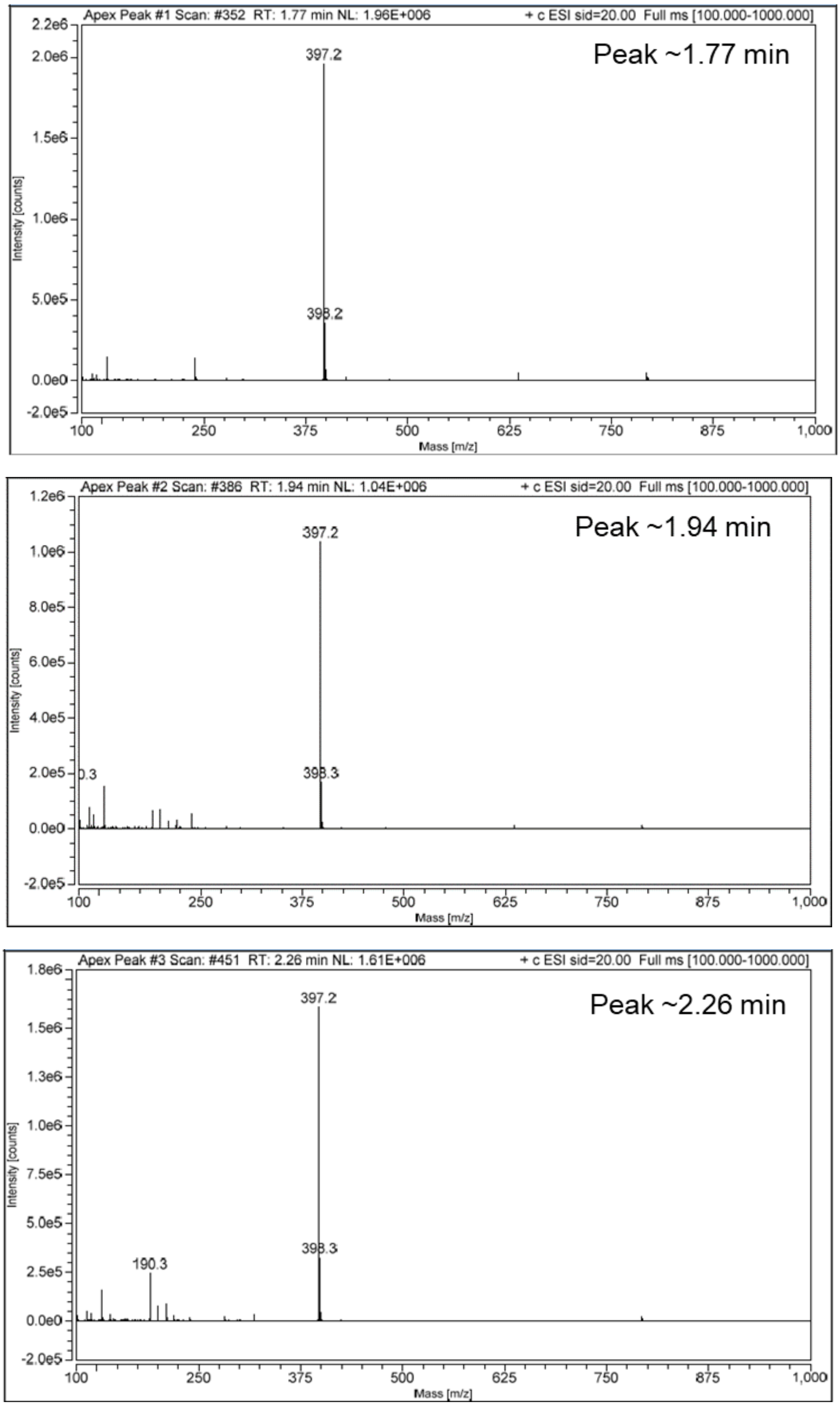
Apex Peak #1 Scan: #352 RT: 1.77 min NL: 1.96E+006
+ c ESI sid=20.00 Full ms [100.000-1000.000]
Peak ~1.77 min
397.2
398.2
Intensity [counts]
Mass [m/z]
Apex Peak #2 Scan: #386 RT: 1.94 min NL: 1.04E+006
+ c ESI sid=20.00 Full ms [100.000-1000.000]
Peak ~1.94 min
397.2
398.3
Intensity [counts]
Mass [m/z]
Apex Peak #3 Scan: #451 RT: 2.26 min NL: 1.61E+006
+ c ESI sid=20.00 Full ms [100.000-1000.000]
Peak ~2.26 min
397.2
398.3
190.3
Intensity [counts]
Mass [m/z]

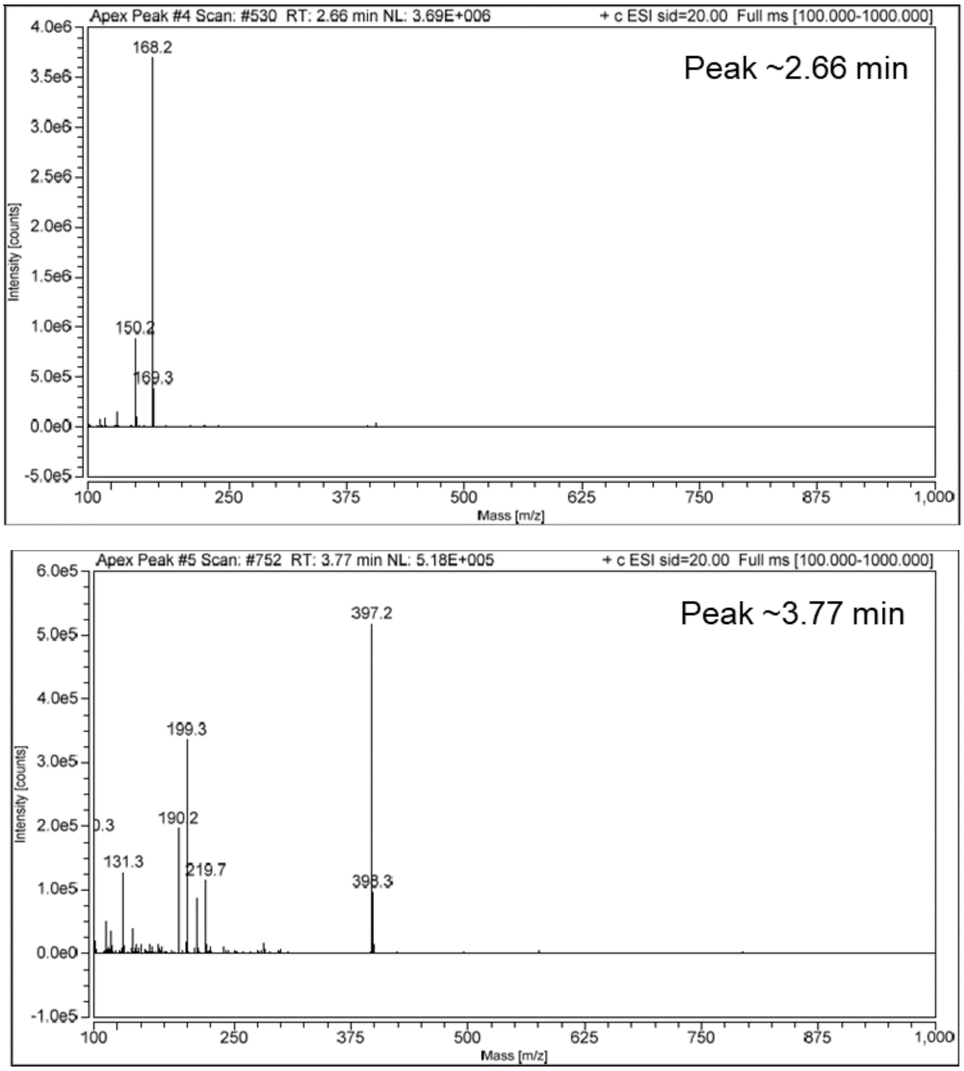

Figure 11: LC-MS results of 1 mM PLP and 1 mM metaraminol in 100 mM HEPES buffer in one batch (pH 7.6) (A) Chromatogram of the reaction mixture with compounds exhibiting retention times of 1.77 min, 1.94 min, 2.26 min, 2.66 min (metaraminol) and 3.77 min . (B) Mass spectra of each extracted peak showing the same m/z of 397.2. With a peak at 2.66 min, an m/z of 168.2 was extracted being most likely the metaraminol.

Quantum mechanics computations of the absorption spectrum of the Pictet-Spengler and oxazolidine product from metaraminol and PLP

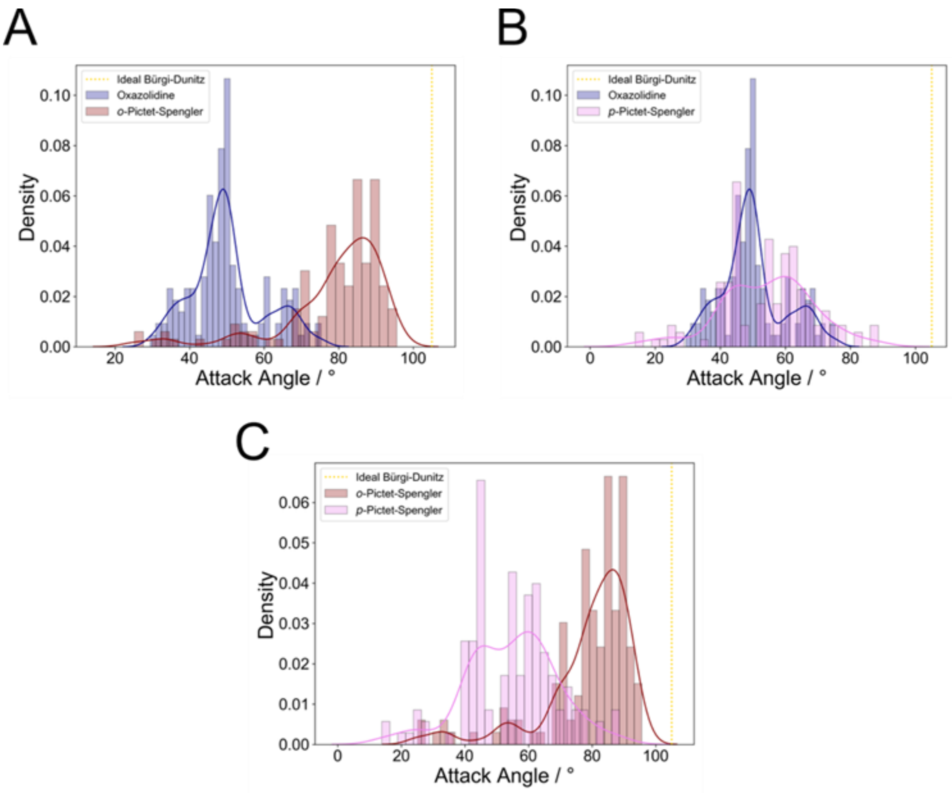

Figure 12: Probability density distribution of the attack angle observed in the CREST ensemble of the Schiff base (Schiff2). The typical ortho-attack angle and para-attack angle distributions for the Pictet-Spengler product formation are shown as dark red and pink histograms, respectively. The typical attack angle for the oxazolidine derivative is shown as blue histograms. The ideal Bürgi-Dunitz angle is shown as a dotted gold vertical line. A) Attack angle comparison between Oxazolidine-like (blue) and o-Pictet-Spengler (red). B) Attack angle comparison between Oxazolidine-like (blue) and p-Pictet-Spengler (pink). C) Attack angle comparison between o-Pictet-Spengler (red) and p-Pictet-Spengler (pink).

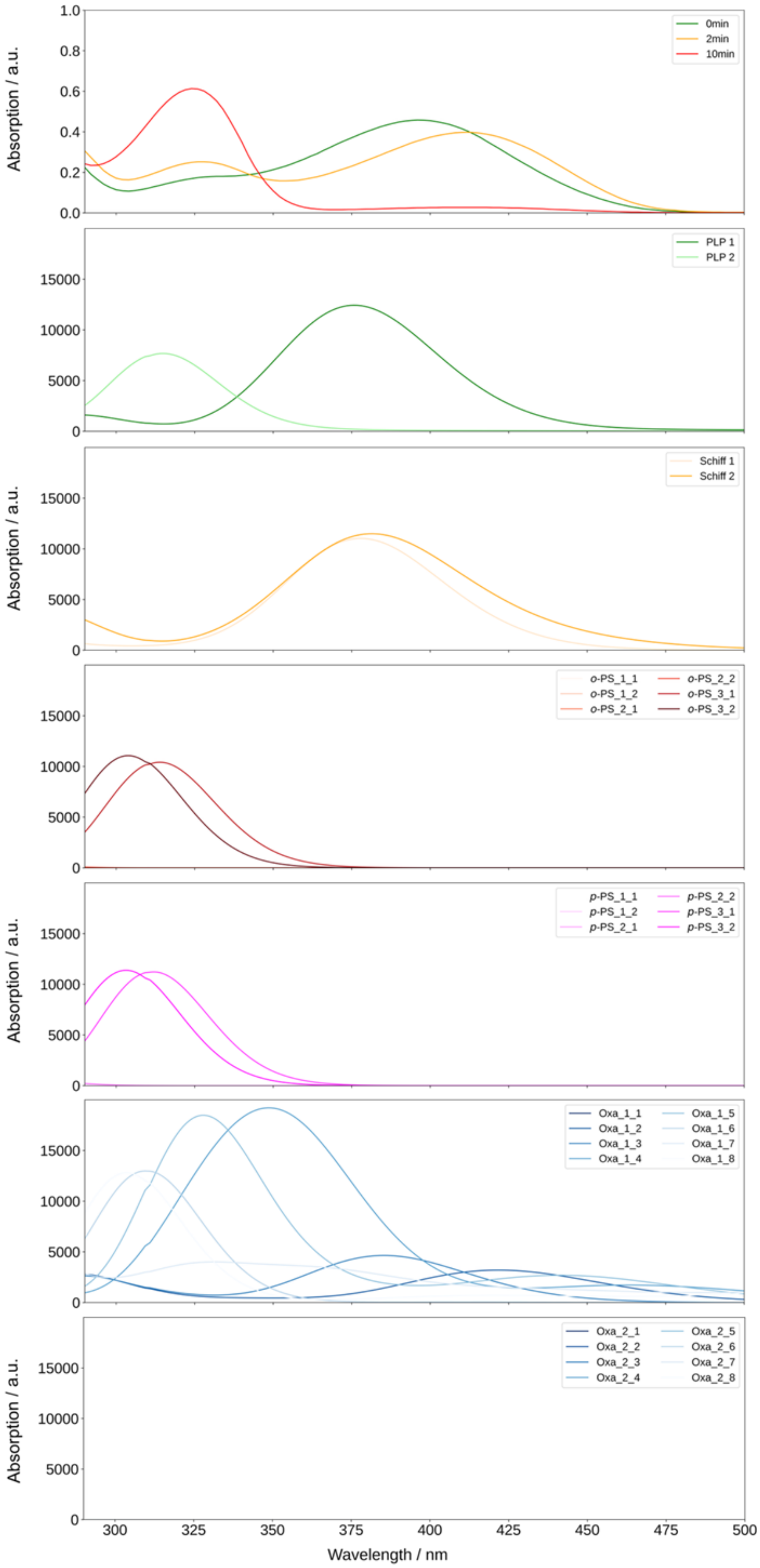

Figure 13: Experimental (top) and computed UV-VIS spectra identify the Pictet-Spengler and the oxazolidine derivatives. The computed spectra are reported for the PLP (in green, two protonation states selected, with PLP1 better fitting to the experimental spectrum; PLP1 protonation was also in agreement with Limbach *et al*., Sharif *et al*. and Chan-Huot *et al*.[51-53] Schiff base spectrum is shown in orange (two protonation states selected, with Schiff2 better fitting to the experimental spectrum). Pictet-Spengler spectra are shown for the ortho-derivative (in brown, three protonation states, two regio-stereoisomers, with o-PS_3_1 better fitting to experimental spectrum) and the para-derivative (in purple, three protonation, two regio-stereoisomers, with p-PS_3_1 better fitting to experimental spectrum). The oxazolidine derivative spectrum is shown for two protonation states (in blue, eight regio-stereoisomer combinations per protonation state, with Oxa_1_6 better fitting to the experimental spectrum).

Table 1: Protonation states and regio-stereoisomers considered in this study. The green-labeled structures are the ones shown in Figure 2B.

**Piridoxal phosphate (PLP)**	
PLP 1	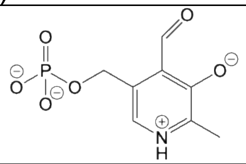
PLP 2	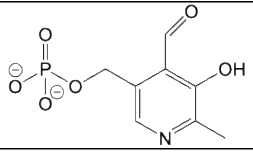
**Schiff base (Schiff)**	
Schiff 1	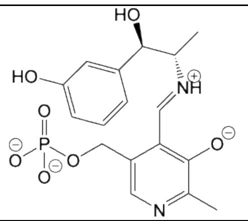
Schiff 2	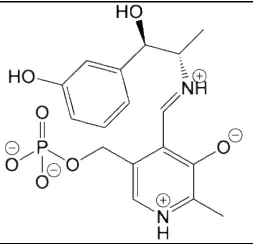
***o*-Pictet-Spengler product (*o*-PS)**	
o-PS_1_1	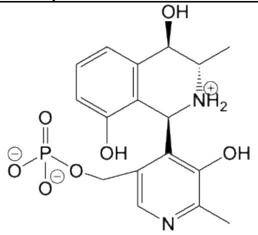
o-PS_1_2	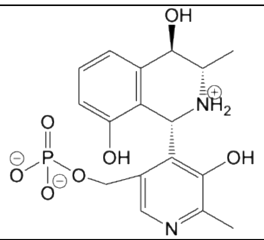
o-PS_2_1	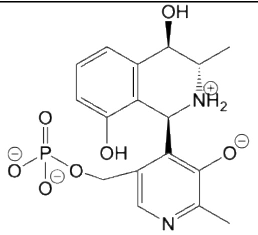
o-PS_2_2	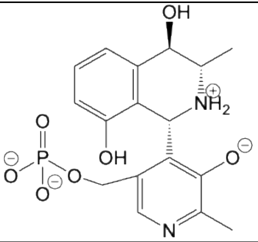

o-PS_3_1	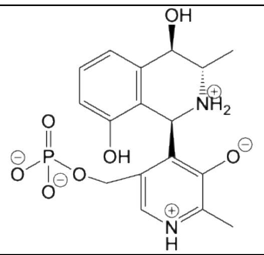
o-PS_3_2	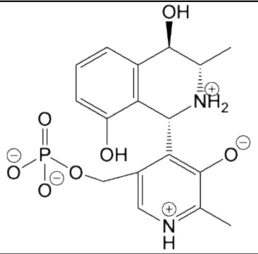
p-Pictet-Spengler product (*p*-PS)	
p-PS_1_1	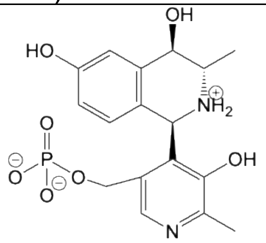
p-PS_1_2	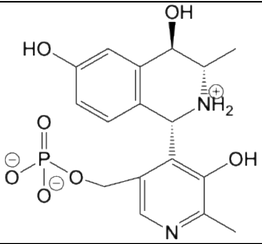
p-PS_2_1	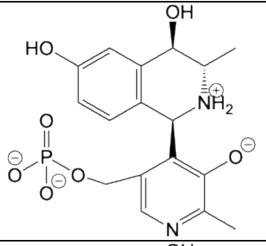
p-PS_2_2	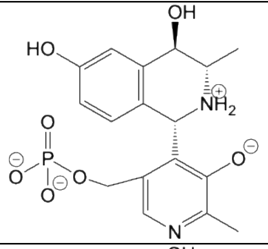
p-PS_3_1	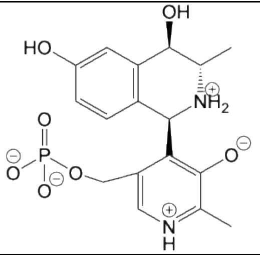

p-PS_3_2	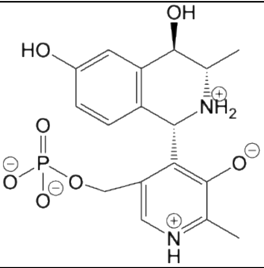
Oxazolidine product (Oxa)	
Oxa_1_1	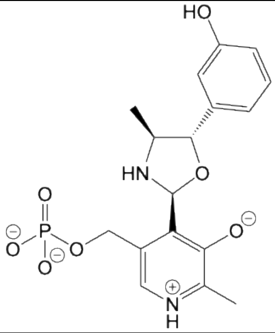
Oxa_1_2	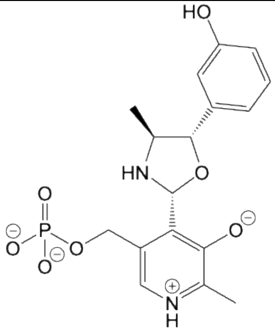
Oxa_1_3	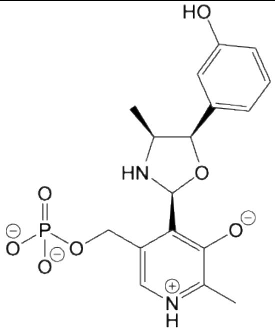
Oxa_1_4	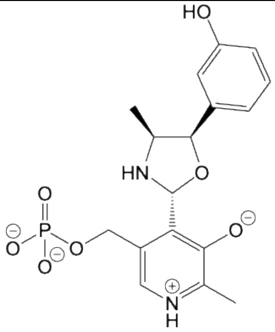

Oxa_1_5	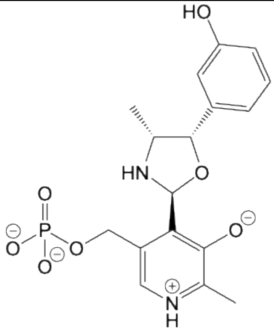
Oxa_1_6	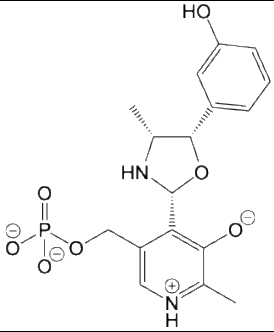
Oxa_1_7	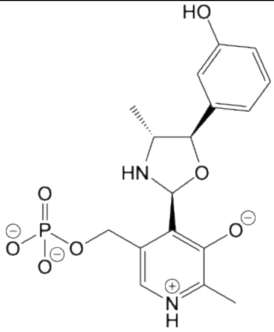
Oxa_1_8	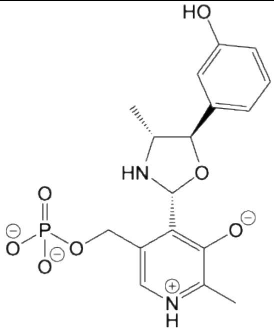
Oxa_2_1	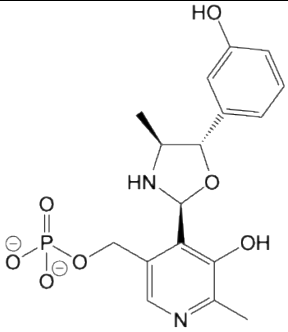

Oxa_2_2	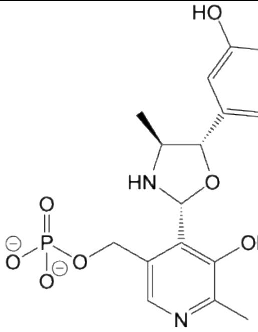
Oxa_2_3	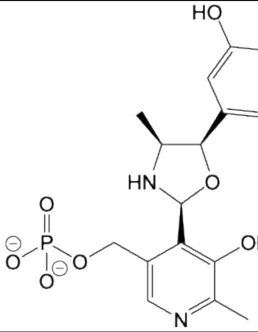
Oxa_2_4	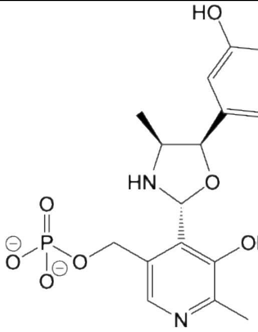
Oxa_2_5	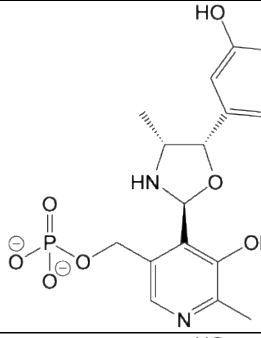
Oxa_2_6	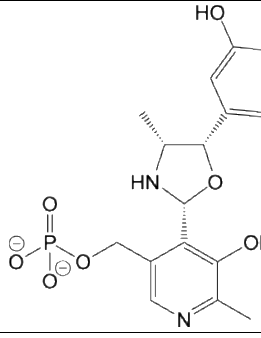

Oxa_2_7	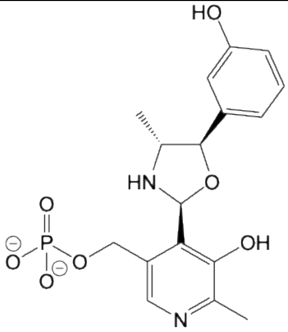
Oxa_2_8	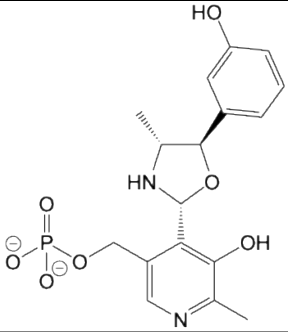

PLP-feed during the *Cv*ATA catalyzed reductive amination of (*R*)-3-OH-PAC towards metaraminol

As PLP is crucial for a functional *Cv*ATA and is therefore commonly supplemented in reaction mixtures containing amine transaminases, we hypothesized that the complex formation between the cofactor and the target product metaraminol could negatively affect the *Cv*ATA function. Thus, only low metaraminol concentrations were yielded. To test this hypothesis, we fed 0.1 mM PLP in the beginning of the reductive amination at three different time points (2.5 h, 4.5 h, 5.5 h). The metaraminol production and the (*R*)-3-OH-PAC depletion over the course of the reaction are depicted in Figure 14.

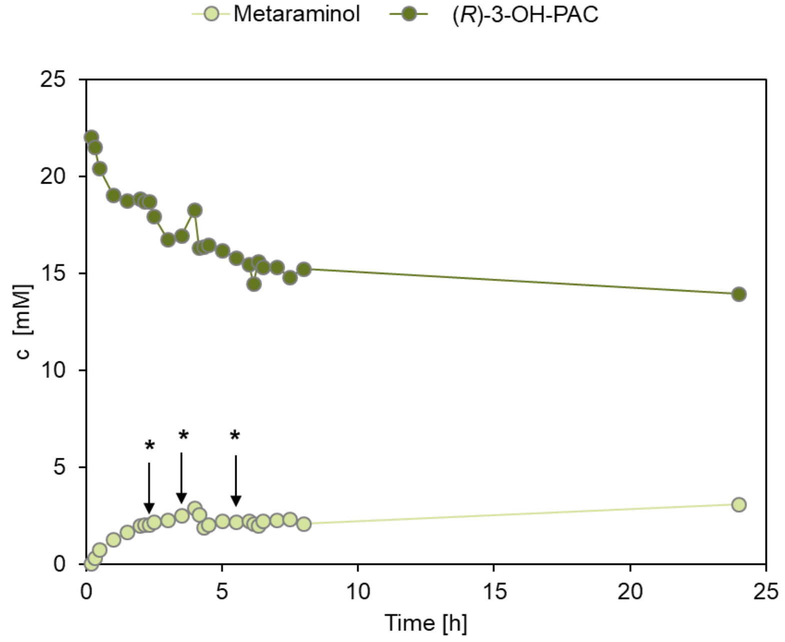

Figure 14: PLP feed during the reductive amination: (*R*)-3-OH-PAC depletion and metaraminol production. Reaction conditions: 0.6 mg·mL^{-1} purified *Cv*ATA (concentration determined via the Bradford method), 20 mM (*R*)-3-OH-PAC, 125 mM isopropylamine, 0.1 mM PLP in 100 mM KPi buffer (pH 7.6). V = 1 mL, T = 30 °C, 1000 rpm. After 2.5 h, 4.5 h and 5.5 h additional 0.1 mM PLP was fed. n = 1.

After each feeding time point, the metaraminol synthesis did not increase. After 5.5 h, 0.1 mM PLP was fed for the last time, and the metaraminol concentration stagnated at 3 mM for the residual reaction time. To conclude, feeding the cofactor did not help in pushing the metaraminol yields. It rather seems that the complexation of PLP and metaraminol does not affect the reaction towards metaraminol in such a way that the reaction yields low product concentrations. The other side-products resulting from (*R*)-3-OH-PAC seem to have a greater impact on the reaction yield.

Side-product formation of the substrate (*R*)-3-OH-PAC

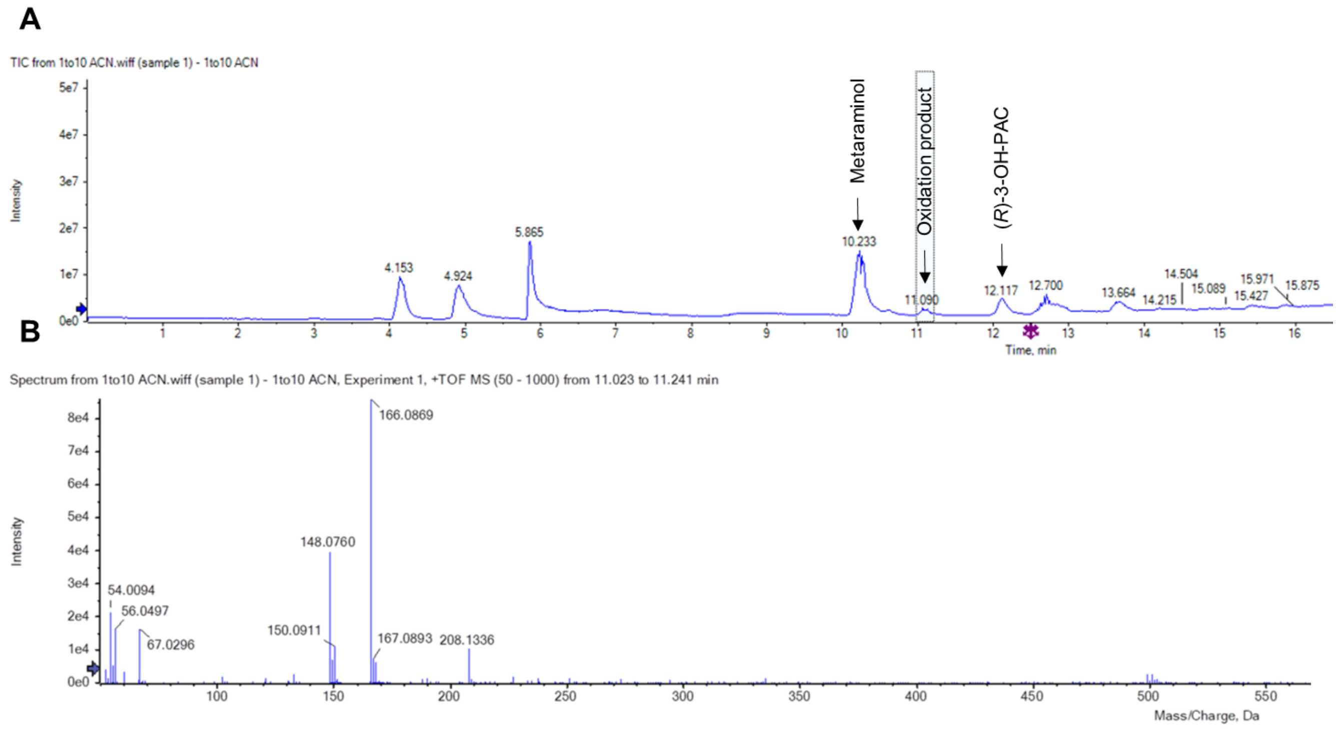

Figure 15: (A) LC chromatogram of the reaction mixture containing 40 mM (*R*)-3-OH-PAC and 250 mM IPA with 1 mM PLP with 20 mg·mL^{1} whole cell *Cv*ATA. Metaraminol elutes after 10.2 min, (*R*)-3-OH-PAC elutes after 12.1 min. (B) Mass spectrum of the unknown peak at 11.1 min, assumed to be 1-(3-hydroxyphenyl)propane-1,2-dione, the oxidation product of (*R*)-3-OH-PAC.

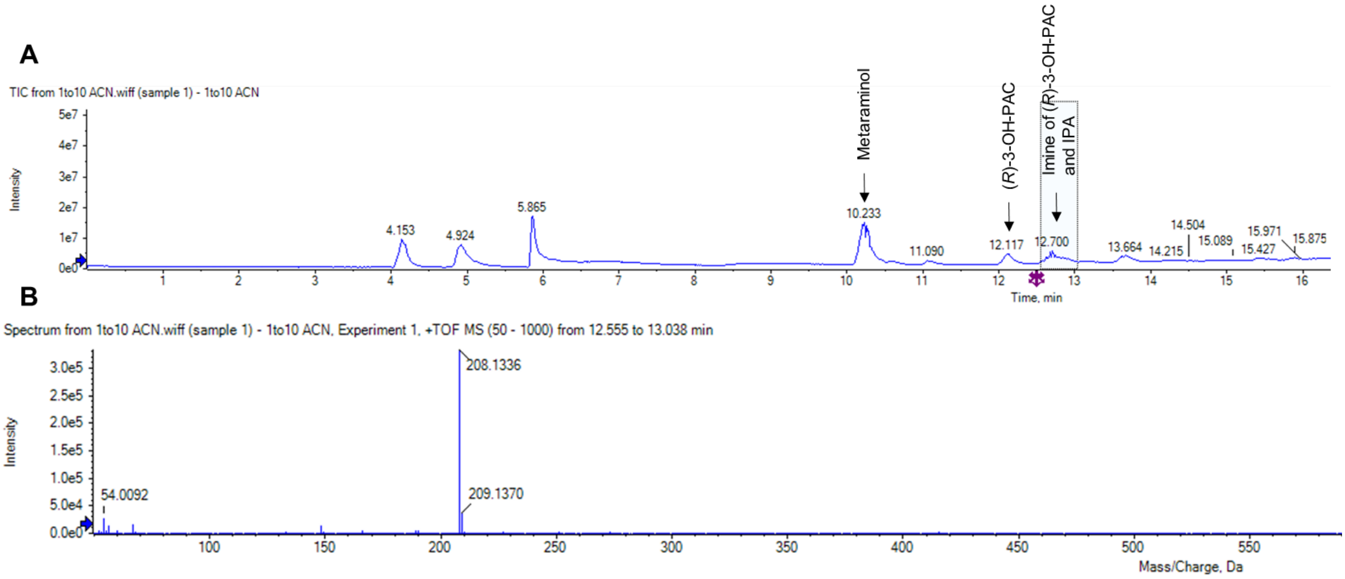

Figure 16: (A) LC chromatogram of the reaction mixture containing 40 mM (*R*)-3-OH-PAC and 250 mM IPA with 1 mM PLP with 20 mg·mL^{1} whole cell *Cv*ATA. Metaraminol elutes after 10.2 min, (*R*)-3-OH-PAC elutes after 12.1 min. The to be analyzed compound elutes after 12.7 min. (B) Mass spectrum of the unknown peak at 12.7 min, assumed to be the imine of (*R*)-3-OH-PAC and IPA.
